# Cell type-specific binding patterns reveal that TCF7L2 can be tethered to the genome by association with GATA3

**DOI:** 10.1186/gb-2012-13-9-r52

**Published:** 2012-09-05

**Authors:** Seth Frietze, Rui Wang, Lijing Yao, Yu Gyoung Tak, Zhenqing Ye, Malaina Gaddis, Heather Witt, Peggy J Farnham, Victor X Jin

**Affiliations:** 1 Department of Biochemistry and Molecular Biology, Norris Comprehensive Cancer Center, University of Southern California, Los Angeles, CA 90089, USA; 2 Department of Chemistry, Lanzhou University, Lanzhou 730000, China; 3 Department of Biomedical Informatics, The Ohio State University, Columbus, OH 43210, USA

## Abstract

**Background:**

The TCF7L2 transcription factor is linked to a variety of human diseases, including type 2 diabetes and cancer. One mechanism by which TCF7L2 could influence expression of genes involved in diverse diseases is by binding to distinct regulatory regions in different tissues. To test this hypothesis, we performed ChIP-seq for TCF7L2 in six human cell lines.

**Results:**

We identified 116,000 non-redundant TCF7L2 binding sites, with only 1,864 sites common to the six cell lines. Using ChIP-seq, we showed that many genomic regions that are marked by both H3K4me1 and H3K27Ac are also bound by TCF7L2, suggesting that TCF7L2 plays a critical role in enhancer activity. Bioinformatic analysis of the cell type-specific TCF7L2 binding sites revealed enrichment for multiple transcription factors, including HNF4alpha and FOXA2 motifs in HepG2 cells and the GATA3 motif in MCF7 cells. ChIP-seq analysis revealed that TCF7L2 co-localizes with HNF4alpha and FOXA2 in HepG2 cells and with GATA3 in MCF7 cells. Interestingly, in MCF7 cells the TCF7L2 motif is enriched in most TCF7L2 sites but is not enriched in the sites bound by both GATA3 and TCF7L2. This analysis suggested that GATA3 might tether TCF7L2 to the genome at these sites. To test this hypothesis, we depleted GATA3 in MCF7 cells and showed that TCF7L2 binding was lost at a subset of sites. RNA-seq analysis suggested that TCF7L2 represses transcription when tethered to the genome via GATA3.

**Conclusions:**

Our studies demonstrate a novel relationship between GATA3 and TCF7L2, and reveal important insights into TCF7L2-mediated gene regulation.

## Background

The *TCF7L2 *(transcription factor 7-like 2) gene encodes a high mobility group box-containing transcription factor that is highly up-regulated in several types of human cancer, such as colon, liver, breast, and pancreatic cancer [1-4]. Although TCF7L2 is sometimes called TCF4, there is a helix-loop-helix transcription factor that has been given the official gene name of *TCF4 *and it is important, therefore, to be aware of possible confusion in the literature. Numerous studies have shown that TCF7L2 is an important component of the WNT pathway [3,5,6]. TCF7L2 mediates the downstream effects of WNT signaling via its interaction with CTNNB1 (beta-catenin) and it can function as an activator or a repressor, depending on the availability of CTNNB1 in the nucleus. For example, TCF7L2 can associate with the members of the Groucho repressor family in the absence of CTNNB1. The WNT pathway is often constitutively activated in cancers, leading to increased levels of nuclear CTNNB1 and up-regulation of TCF7L2 target genes [3]. In addition to being linked to neoplastic transformation, variants in TCF7L2 are thought to be the most critical risk factors for type 2 diabetes [7-10]. However, the functional role of TCF7L2 in these diseases remains unclear. One hypothesis is that TCF7L2 regulates its downstream target genes in a tissue-specific manner, with a different cohort of target genes being turned on or off by TCF7L2 in each cell type. One way to test this hypothesis is to identify TCF7L2 target genes in a diverse set of cell types.

Previous studies have used genome-wide approaches to identify TCF7L2 target genes in human colon cancer cells [11,12] and, more recently, chromatin immunoprecipitation sequencing (ChIP-seq) analysis of TCF7L2 was reported in hematopoietic cells [13]. In addition, TCF7L2 binding has been studied in rat islets and rat hepatocytes [14,15]. However, to date no one study has performed comparative analyses of genome-wide binding patterns of TCF7L2 in diverse human cell types. We have now conducted ChIP-seq experiments and comprehensively mapped TCF7L2 binding loci in six human cell lines. We identified datasets of common and cell-specific TCF7L2 binding loci and a set of predicted TCF7L2-regulated enhancers (by comparing the TCF7L2 peak locations with ChIP-seq data for the active enhancer marks H3K4me1 (histone H3 monomethylated on lysine 4) and H3K27Ac (histone H3 acetylated on lysine 27)). We also predicted bioinformatically and confirmed experimentally that TCF7L2 co-localizes with cell type-specific factors. Finally, we showed that GATA3 (GATA binding protein 3), which co-localizes with TCF7L2 in MCF7 breast cancer cells, is required for recruitment of TCF7L2 to a subset of binding sites. Our studies reveal new insights into TCF7L2-mediated gene regulation and suggest that cooperation with other factors dictates different roles for TCF7L2 in different tissues.

## Results

### Defining TCF7L2 genomic binding patterns

To identify TCF7L2 binding loci in a comprehensive manner, we performed ChIP-seq using an antibody to TCF7L2 and profiled six human cell types, including colorectal carcinoma cells (HCT116), hepatocellular carcinoma cells (HepG2), embryonic kidney cells (HEK293), mammary gland adenocarcinoma cells (MCF7), cervical carcinoma cells (HeLa), and pancreatic carcinoma cells (PANC1). We chose these particular cell lines because TCF7L2 has been associated with these types of cancers and because all of these cells have various data sets associated with them as part of the ENCODE project. The *TCF7L2 *gene has 17 exons, including 5 exons that are alternatively spliced in different tissues [2,16-20]. Alternative splicing produces two major isoforms of TCF7L2 in most cells, a cluster of isoforms of approximately 79 kDa and a cluster of isoforms of approximately 58 kDa. All of these isoforms contain the DNA binding domain, the CTNNB1 binding domain, the Groucho binding domain, and the nuclear localization signal. However, the CtBP (C-terminal binding protein) binding domain is encoded at the carboxyl terminus and is missing in the 58 kDa isoform [21,22]. The two major isoforms are found at similar ratios in the six cell lines that we analyzed (Additional file 1). For all cell types, we performed duplicate ChIP-seq assays using chromatin from two different cell culture dates (see Additional file 2 for details concerning all ChIP-seq experiments and information on how to access the data). To ensure that our data were of high quality and reproducible, we called peaks [11,23,24] and then compared the peak sets using the ENCODE overlap rules (Additional file 3); all datasets had a high degree of reproducibility (Additional file 4). We next combined the reads for each replicate experiment and called TCF7L2 peaks for each cell type, identifying tens of thousands of peaks in each cell type (Table 1; see Additional file 5 for lists of all TCF7L2 binding sites in each cell type and Additional file 6 for a summary of the peak characteristics for each cell type). We used a saturation analysis strategy (Additional file 3) to demonstrate that the depth of sequencing of the ChIP samples was sufficient to identify the majority of TC7L2 binding sites in each cell type (Additional file 7).

**Table 1:** TCF7L2 binding sites and target genes

	Total peaks	Nearest genes	Peaks per gene
HCT116	30,259	10,910	2.8
HEK293	24,457	6,254	3.9
HepG2	27,912	7,899	3.5
MCF7	27,721	6,934	4.0
HeLa	52,810	11,334	4.7
PANC1	31,744	9,438	3.4
Common	1,864	1,287	1.4
Total unique	116,270	14,193	8.2

Peaks were called using the BELT program on the merged duplicate datasets for each cell type. Reported as the 'Total peaks' is the total number of TCF7L2 binding sites in each cell line, as well as the total number of unique peaks when all six cell lines are considered together and the peaks identified as being common to all six cell lines. To determine the number of 'Nearest genes', the RefSeq gene nearest each TCF7L2 binding site was identified and duplicates were removed (since TCF7L2 has multiple binding sites near many genes), and the resultant number is indicated as the total number of target genes for that peak set.

**Figure 1:**
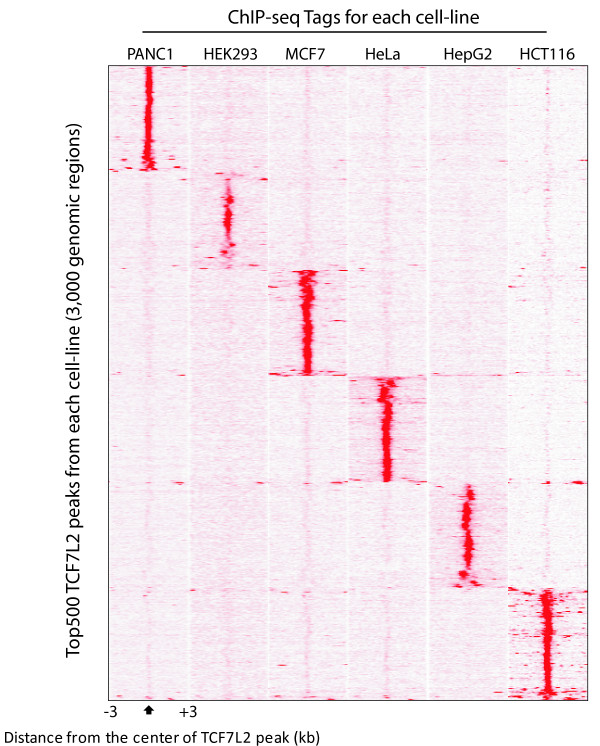
**ChIP-seq analysis of TCF7L2 in six different human cell lines**. Shown is the distribution of TCF7L2 binding within ±3 kb windows around distinct genomic regions (n = 3,000) bound by TCF7L2 in a given cell type. ChIP-seq tags for each cell line were each aligned with respect to the center of the combined top 500 peaks from each dataset and clustered by genomic position.

We next determined if the TCF7L2 binding sites identified in each cell type are unique to that cell type or if TCF7L2 binds to the same locations in different cells. We first performed two-way comparisons of the peaks from all six cell types and found that the overlaps ranged from a low of 18% of HepG2 sites being present in the HEK293 peak set to a high of 46% of the HCT116 sites being present in the PANC1 peak set. These low overlaps suggested that each cell type contributes a unique set of peaks. To demonstrate the cell type specificity of binding of TCF7L2, the top 500 binding sites were selected from the ChIP-seq datasets from each of the 6 cell types (a total of 3,000 peaks). Then, the sequenced tags in all 6 datasets corresponding to the genomic regions spanning ±3 kb from the center of each of the combined 3,000 peaks were clustered with respect to these genomic regions (Figure 1). This analysis demonstrates a clear cell type-specificity in the top-ranked TCF7L2 binding sites. We note that one characteristic of cancer cell lines is that they often have extensive genomic amplifications. Peak calling programs (such as the ones used in our analyses) that use input DNA from the specific cancer cell can help to prevent many false positive peaks that would otherwise rise to the top of the peak list due to the fact that the amplified regions are 'over-sequenced' in comparison to the rest of the genome. However, it is difficult to completely account for amplifications. Therefore, to ensure that the cell type specificity that we observed was not due to TCF7L2 peaks in amplified regions, we used our peak-calling program Sole-search to identify all genomic amplifications in the six cancer cell lines (Additional file 8). Then, we identified the TCF7L2 peaks that are in the amplified regions in each cell line (Additional file 9); all peaks from amplified regions were removed from the peak lists prior to the analysis shown in Figure 1. In total, we found that each cell type had more than 10,000 TCF7L2 binding sites that were not found in any of the sets of called peaks for the other 5 cell types (see Additional file 10 for lists of cell type-specific TCF7L2 binding sites). Of course, it is possible that some sites that appear to be cell type-specific are actually very small peaks in another cell type and fall below the cutoff used in our analyses. Overall, we identified 116,270 non-redundant TCF7L2 binding sites when the datasets from all 6 cell lines are combined. Only 1,864 TCF7L2 binding loci were common to all 6 cell lines, suggesting that TCF7L2 may play an important, yet distinct, role in different cells.

**Figure 2:**
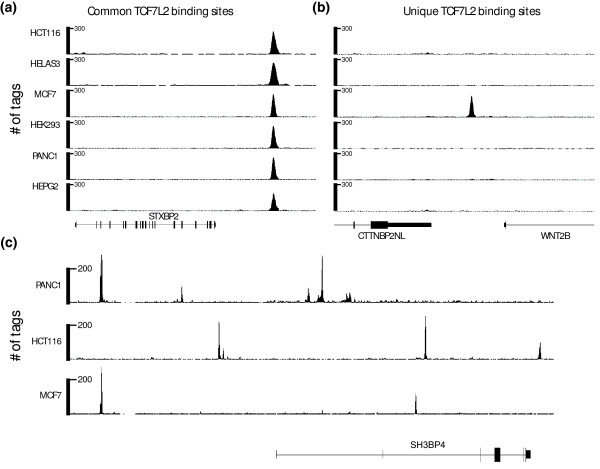
**Cell type-specific binding of TCF7L2**. **(a,b) **The ChIP-seq binding patterns of TCF7L2 are compared in six cell lines, demonstrating both common peaks (a) and cell type-specific binding (b). **(c) **The ChIP-seq binding patterns of TCF7L2 near and within the SH3BP4 locus is shown for three cell lines. The number of tags reflecting the ChIP enrichments are plotted on the y-axis; the chromosomal coordinates (hg19) shown are: (a) chr19:7,701,591-7,718,750; (b) chr1:112,997,195-113,019,766; and (c) chr2:235,767,270-235,974,731.

To confirm the cell-specific TCF7L2 binding loci that we observed in the ChIP-seq data, we chose a set of three targets identified to be cell type-specific for each of the six cell lines, three common targets, and three negative regions not bound by TCF7L2 in any cell line and performed quantitative ChIP-PCR (ChIP-qPCR) using DNA isolated from ChIP samples that were distinct from the samples used for ChIP-seq (Additional file 11). The common targets were bound by TCF7L2 in all samples, whereas the negative controls showed very low enrichment in all samples. In general, regions identified as cell type-specific showed the greatest enrichment for TCF7L2 binding in that corresponding cell line (for example, the PANC1-specific sites showed very high enrichment in ChIP samples from PANC1 cells, low enrichment in samples from HepG2, HeLa, and HCT116 cells, and no enrichment in samples from HEK293 or MCF7 cells). Thus, ChIP-qPCR confirms the specificity of targets identified in the ChIP-seq data in each cell line. Examples of common and cell type-specific TCF7L2 binding sites are shown in Figure 2.

To determine the potential set of genes regulated by TCF7L2 in each cell type, we identified the closest annotated gene to each TCF7L2 binding site in the six different cell types and the closest annotated gene to the set of 1,864 common TCF7L2 binding sites. The number of target genes (as defined by the nearest gene to a TCF7L2 binding site) ranged from approximately 6,000 to 11,000 in the different cell lines (Table 1). In addition, we also observed that the number of target genes in each cell line was considerably less than the number of TCF7L2 binding sites, demonstrating that TC7L2 binds to multiple locations near each target gene (Table 1). Although less than 2% (1,864 of 116,270 peaks) of the total number of peaks were commonly bound by TCF7L2 in all 6 cell lines, 9% of target genes were common to all 6 cell lines (1,287 of 14,193 genes). This indicates that TCF7L2 regulates certain genes in different cell types using different binding sites. For example, there are 12 TCF7L2 binding sites near the SH3BP4 gene, but these sites are different in MCF7, HCT116, and PANC1 cells (Figure 2c).

**Figure 3:**
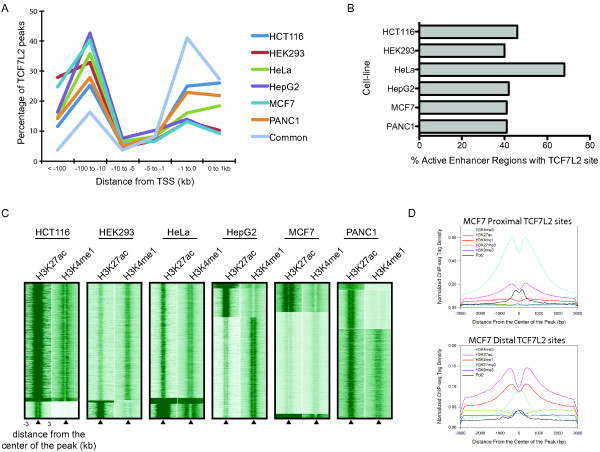
**TCF7L2 binding sites are distal and enriched for active enhancer histone marks**. **(a) **Shown for the TCF7L2 binding sites in the six cell types and for the 1,864 peaks commonly bound in all six cells is the percentage of TCF7L2 binding sites in different genomic regions (hg19) relative to the nearest transcription start site (TSS). **(b) **The percentage of active enhancer regions containing a TCF7L2 binding site; active enhancers were defined by taking the regions that have an overlap of H3K4me1 and H3K27ac ChIP-seq peaks for the given cell line. **(c) **Heatmaps of the ChIP-Seq tags for H3K27ac and H3K4me1 at TCF7L2-bound regions (±3 kb windows around the center of all TCF7L2 peaks) for each cell line were generated by k-means cluster analysis. **(d) **The average RNA polymerase II and histone modification profiles of MCF7 cells are shown for the ±3 kb windows around the center of TCF7L2 peaks identified as proximal to RefSeq genes (upper graph) or distal to RefSeq genes (lower graph).

The binding patterns shown in Figure 2c indicate that TCF7L2 does not necessarily bind to promoter regions, but rather binds to a variety of genomic locations near or within the SH3BP4 locus. To evaluate the global distribution of TCF7L2 binding loci in each cell line, we plotted the percentage of TCF7L2 sites versus their distance to the nearest transcription start site. Even though TCF7L2 binds to different sites in the different cell lines, the trend for the distribution of TCF7L2 target loci is the same for each cell line (Figure 3a). Although some of the TCF7L2 binding sites are within 1 kb of a transcription start site, most of the sites are located at distances greater than 10 kb from a start site. However, we did find that the common sites to which TCF7L2 is bound in all six cell lines are more enriched near the 5' of a gene than are the other sites (Figure 3a). A detailed analysis of the TCF7L2 binding sites, including the location of each site relative to the transcription start site of the nearest gene for all peaks in each of the six cell lines can be found in Additional file 5.

### TCF7L2 binds to enhancer regions

The fact that TCF7L2 can bind to regions far from core promoters suggested that TCF7L2 might bind to enhancers. Recent studies have shown that enhancers can be identified by enrichment for both the H3K4me1 and H3K27Ac marks [25-27]. To determine if the regions bound by TCF7L2 are also bound by these modified histones, we performed ChIP-seq experiments in PANC1, HEK293, HCT116 and MCF7 cells using antibodies that specifically recognize histone H3 only when it is monomethylated on lysine 4 or when it is acetylated on lysine 27; we also used H3K4me1 and H3K27Ac data for HeLa and HepG2 cells from the ENCODE project. Duplicate ChIP-seq experiments were performed using two different cultures of cells for each cell type, peaks were called individually to demonstrate reproducibility (Additional file 4), the reads were merged and a final peak set for both H3K4me1 and H3K27Ac was obtained. We then identified predicted active enhancers as regions having both H3K4me1 and H3K27Ac and determined the percentage of the TCF7L2 sites that have either or both of the modified histones (Table 2). We found that, for most cells, the majority of TCF7L2 sites co-localized with H3K4me1 and H3K27Ac. However, a smaller percentage of the TCF7L2 sites in MCF7 cells co-localized with active enhancers. Heatmaps of the tag density of the histone ChIP-seq experiments for each cell line relative to the center of the TCF7L2 peak locations are shown in Figure 3c. Although most TCF7L2 binding sites show robust levels of both marks, the TCF7L2 sites in MCF7 cells again show a smaller percentage of sites having high levels of the modified histones. To determine if the TCF7L2 binding sites in MCF7 cells correspond to sites bound by histone modifications associated with transcriptional repression, we performed duplicate ChIP-seq analysis using antibodies to H3K9me3 (histone H3 trimethylated on lysine 9) and H3K27me3 (histone H3 trimethylated on lysine 27); we also used H3K4me3 (histone H3 trimethylated on lysine 4) and RNA polymerase II ChIP-seq data from the ENCODE project. As shown in Figure 3d, neither the proximal nor distal TCF7L2 binding sites show high levels of H3K9me3 or H3K27me3.

**Table 2:** TCF7L2 binds to enhancer regions

Cell line	Percentage of TCF7L2 peaks at H3K4me1 sites	Percentage of TCF7L2 peaks at H3K27Ac sites	Percentage of TCF7L2 peaks at active enhancers (H3K4me1 and H3K27Ac)
HCT116	68	72	57
HepG2	84	59	56
PANC1	63	74	55
HeLa	72	58	53
MCF7	34	46	25

TCF7L2 peaks were overlapped with called peaks for H3K27Ac or H3K4me1 or both H3K27Ac and H3K4me1 in the same cell line. The percentage of TCF7L2 peaks in each category is indicated.

To further investigate the role of TCF7L2 in cell type-specific enhancers, we determined the percentage of active enhancers in each of the six cell types (that is, genomic regions bound by both H3K4me1 and H3K27Ac) that are also bound by TCF7L2. We found that more than 40% of all enhancers in the different cell lines are occupied by TCF7L2 (Figure 3b). These results indicate that TCF7L2 ChIP-seq data identify many of the active enhancers in a given cell type and suggest that TCF7L2 may play a critical role in specifying the transcriptome in a variety of cancer cells. An example of TCF7L2 binding to sites marked by H3K4me1 and H3K27Ac in HepG2 cells is shown in Additional file 12; TCF7L2 does not bind to this same site in HeLa cells and these sites are also not marked by the modified histones in HeLa cells.

### Motif analysis of genomic regions bound by TCF7L2

To investigate the predominant motifs enriched in TCF7L2 binding sites, we applied a *de novo *motif discovery program, ChIPMotifs [28,29], to the sets of TCF7L2 peaks in each cell type. We retrieved 300 bp for each loci from the top 1,000 binding sites in each set of TCF7L2 peaks and identified the top represented 6-mer and 8-mer (Additional file 13). For all cell lines, the same 6-mer (CTTTGA) and 8-mer (CTTTGATC) motif was identified (except for HCT116 cells, for which the 8-mer was CCTTTGAT). These sites are almost identical to the Transfac binding motifs for TCF7L2 (TCF4-Q5:SCTTTGAW) and for the highly related family member LEF1 (LEF1-Q2:CTTTGA) and to experimentally discovered motifs in previous TCF7L2 ChIP-chip and ChIP-seq data [11,30]. These motifs are present in a large percentage of the TCF7L2 binding sites. For example, more than 80% of the top 1,000 peaks in each dataset from each cell type contain the core TCF7L2 6-mer W1 motif, with the percentage gradually dropping to approximately 50% of all peaks (Additional file 14).

**Figure 4:**
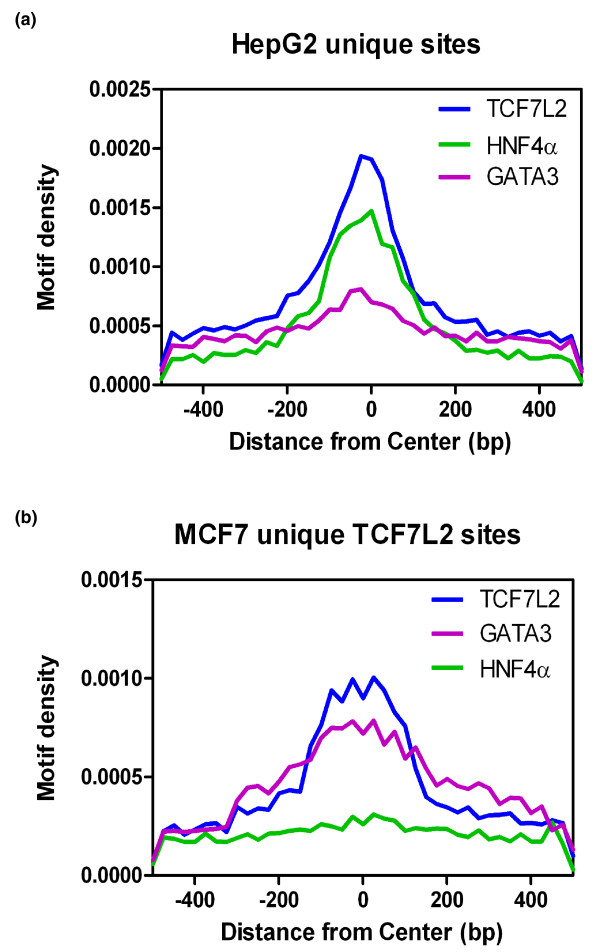
**Association of other motifs with TCF7L2 binding sites**. **(a,b) **TCF7L2 binding sites unique to HepG2 cells (a) or MCF7 cells (b) were analyzed for the indicated motifs; the position of each motif is plotted relative to the center of the TCF7L2 binding site.

Because the TCF7L2 motif is present in all the cell lines at the same genomic locations, but TCF7L2 binds to different subsets of the TCF7L2 motifs in the different cell lines, this suggests that a cell type-specific factor may help to recruit and/or stabilize TCF7L2 binding to specific sites in different cells. Also, as shown above, TCF7L2 binds to enhancer regions, which are typified by having binding sites for multiple factors. To test the hypothesis that TCF7L2 associates with different transcription factor partners in different cell types, we identified motifs for other known transcription factors using the program HOMER [31]. For these analyses, we used the subset of TCF7L2 binding sites that were specific to each of the six different cell types. The top four significantly enriched non-TCF7L2 motifs for each dataset are shown in Table 3; many of these motifs correspond to binding sites for factors that are expressed in a cell type-enriched pattern. To assess the specificity of the identified motifs with respect to TCF7L2 binding, we chose one motif specific to HepG2 TCF7L2 binding sites (hepatocyte nuclear factor (HNF)4α) and one motif specific to MCF7 TCF7L2 binding sites (GATA3) and plotted motif densities in the HepG2 cell type-specific TCF7L2 peaks (Figure 4a) and the MCF7 cell type-specific TCF7L2 peaks (Figure 4b). In HepG2 cells, the HNF4α motif, but not the GATA3 motif, is highly enriched at the center of TCF7L2 binding regions. In contrast, in MCF7 cells the GATA3 motif, but not the HNF4α motif, is highly enriched at the center of TCF7L2 binding regions.

**Table 3:** TCF7L2 cell type-specific modules

Cell line	Top four co-localized motifs
HCT116	AP1, CTCF, NF-E2, SP1
HEK293	HOXC9, CDX2, PDX1, FOXA1
HepG2	HNF4α, FOXA2, ERRα, PPARγ
HeLa-S3	ERG, MAZ, CEBP, NF-E2
MCF7	GATA3, AP2, TEAD, AP1
PANC1	CDX2, FOXA1, TEAD, RUNX2

The sets of TCF7L2 binding sites specific for each cell line (not found in any of the other five cell lines) were analyzed using HOMER. The top four most highly enriched motifs (as determined by *P*-value), not including the TCF7L2 motif, are shown for each cell line.

### TCF7L2 co-localizes with HNF4α and FOXA2 in HepG2 cells

**Figure 5:**
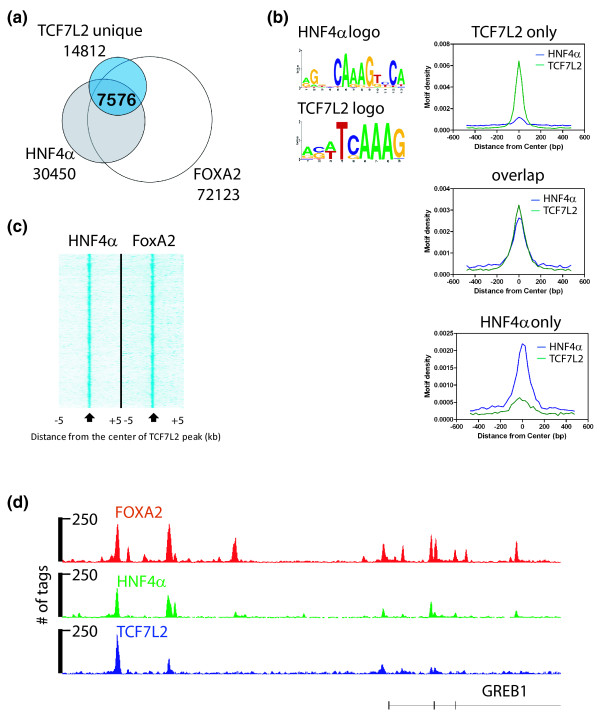
**Association of TCF7L2 and HNF4α in HepG2 cells**. **(a) **HNF4α and FOXA2 ChIP-seq data were downloaded from the UCSC genome browser, and peaks were called and overlapped with the HepG2 cell type-specific TCF7L2 peaks. **(b) **Peaks bound only by HNF4α, only by TCF7L2, or by both factors were analyzed for the presence of HNF4α and TCF7L2 motifs. **(c) **For the set of 7,576 peaks bound by all three factors, the location of the HNF4α and FOXA2 peaks were plotted relative to the center of the TCF7L2 peak. **(d) **A comparison of TCF7L2, HNF4α, and FOXA2 binding patterns near the GREB1 locus is shown. The hg19 genomic coordinates are chr2:11,636,208-11,708,654. The number of tags reflecting the ChIP enrichments is plotted on the y-axis.

To validate the co-localization of TCF7L2 with factors binding to the identified motifs in HepG2 cells, we obtained ChIP-seq data for HNF4α and FOXA2 (forkhead box a2) from the ENCODE Consortium and overlapped the peak sets with the set of TCF7L2 peaks specific for HepG2 cells (Figure 5a). We found that approximately 50% of all HepG2-unique TCF7L2 sites are shared by HNF4α and FOXA2. The sites bound only by HNF4α, only by TCF7L2, or by both factors were analyzed for enrichment of the HNF4α and TCF7L2 motifs (Figure 5b). We found that the motifs were only enriched in the set of peaks specifically bound by each factor. For example, the sites bound only by TCF7L2 but not by HNF4α have TCF7L2 motifs but do not have HNF4α motifs (and vice versa). However, sites bound by both TCF7L2 and HNF4α have motifs for both factors. These results indicate that the HNF4α motif was not identified simply due to its sequence being similar to the TCF7L2 motif and suggest that both factors bind directly to the DNA at the co-localizing sites. We next plotted the location of the experimentally determined HNF4α and FOXA2 sequence tags relative to the center of the TCF7L2 binding site in the set of 7,576 peaks bound by all three factors. As shown in Figure 5c, both HNF4α and FOXA2 localize near the center of the TCF7L2 binding sites. An example of the binding patterns of all three factors at the GREB1 locus is shown in Figure 5d. These results support the hypothesis that HNF4α and FOXA2 may be involved in specifying a portion of the binding of TCF7L2 in liver cells.

### GATA3 is required for TCF7L2 recruitment to a subset of sites in MCF7 cells

**Figure 6:**
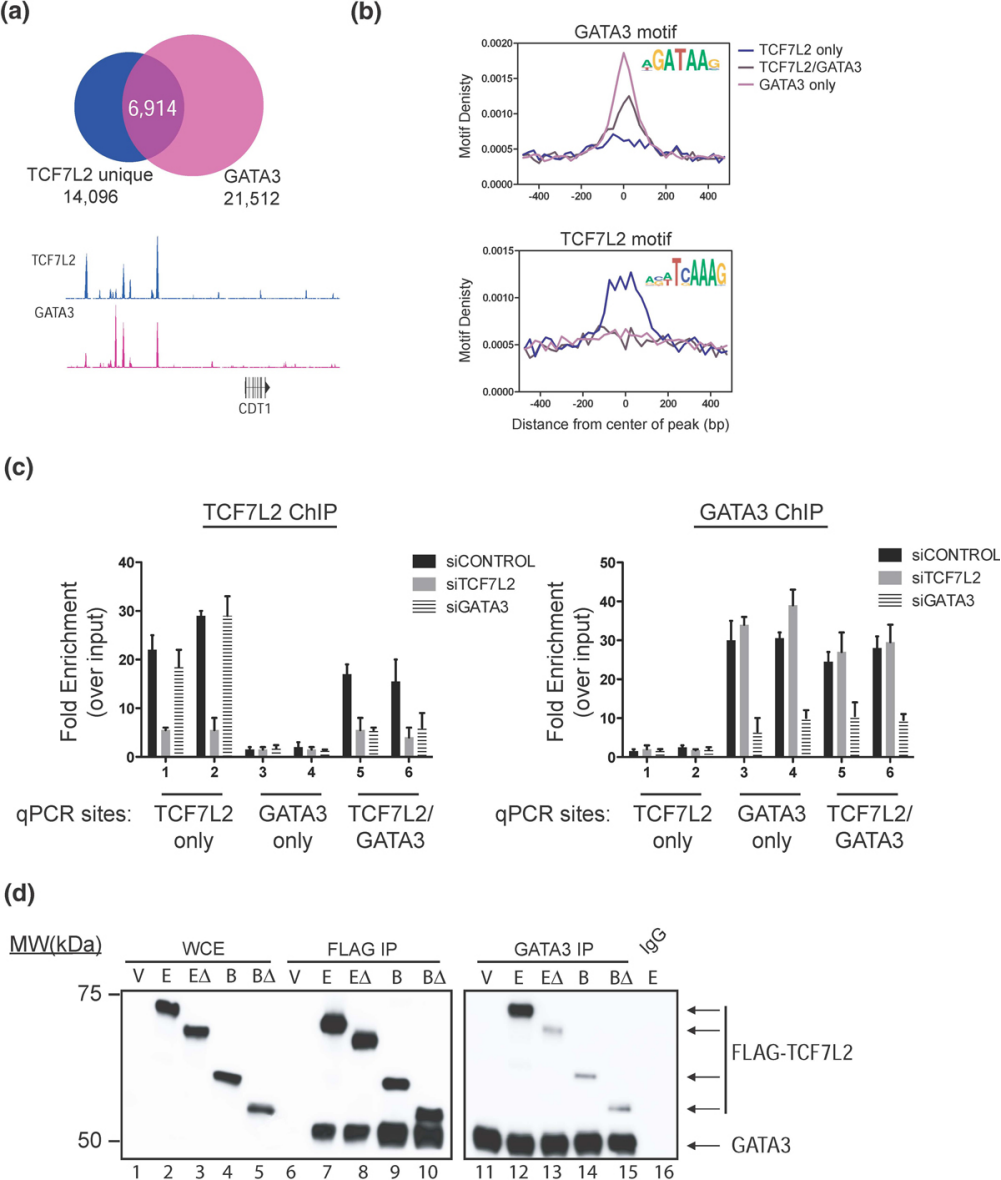
**Association of TCF7L2 and GATA3 in MCF7 cells**. **(a) **GATA3 ChIP-seq in MCF7 cells was performed, and peaks were called and then overlapped with the MCF7 cell type-specific TCF7L2 peaks; a comparison of TCF7L2 and GATA3 binding patterns near the CDT1 locus is shown. The hg19 genomic coordinates are chr16:88,861,964-88,880,233. **(b) **Peaks bound only by GATA3, only by TCF7L2, or by both factors were analyzed for the presence of GATA3 and TCF7L2 motifs. The GATA3 motif is found in sites bound by GATA3 only and in sites bound by both factors, whereas the TCF7L2 motif is found only in the sites bound only by TCF7L2 and not in the sites bound by both factors. **(c) **Depletion of GATA3 results in loss of TCF7L2 occupancy at sites bound by TCF7L2 and GATA3 sites but not at sites only bound by TCF7L2. MCF7 cells were transfected with siRNAs specific for TCF7L2 or GATA3 or control siRNAs. ChIP-qPCR assays were performed using antibodies specific for TCF7L2 (left panel) or GATA3 (right panel) using primers specific for peaks bound only by GATA3, only by TCF7L2, or by both factors. Shown are ChIP-qPCR results performed in triplicate and plotted with the standard error of two independent experiments. **(d) **Co-immunoprecipitation of endogenous GATA3 and FLAG-tagged TCF7L2 constructs from MCF7 cells. The left panel analyzes whole-cell extracts (WCE) and FLAG immunoprecipitation (FLAG IP) eluates from MCF7 cells transfected with the indicated FLAG-tagged plasmids; the membrane was incubated with both anti-FLAG and anti-GATA3 antibodies. Note that the GATA3 signal in input WCE extracts is quite weak and can generally only be visualized after concentration by immunoprecipitation. The right panel is a separate blot prepared in the same way (using the GATA antibody for immunoprecipitation), but does not include the WCE extracts. V, vector control; E, full length TCF7L2; EΔ, TCF7L2 lacking the amino terminus; B, TCF7L2 isoform lacking the carboxyl terminus; BΔ, TCF7L2 isoform lacking the amino and carboxyl termini.

We next examined the relationship between GATA3 and TCF7L2 binding in MCF7 cells. We performed duplicate ChIP-seq experiments for GATA3 in MCF7 cells, called peaks, and then determined the overlap of GATA3 peaks with the TCF7L2 peaks in MCF7 cells (Figure 6a). We found that nearly half of all MCF7-unique TCF7L2 sites are bound by GATA3 (49%); an example of the binding patterns of both factors at the CDT1 locus is shown in Figure 6a. The observation that two factors bind to the same location in the genome could be a result of both factors binding to the same (or nearby) site at the same time or could be due to one factor binding to the genomic region in one cell with the other factor binding to that same region in a different cell in the population. To address these possibilities, we performed motif analyses, co-immunoprecipitations, and knockdown experiments. The sites bound only by GATA3, only by TCF7L2, or by both factors were analyzed for the enrichment of the GATA3 and TCF7L2 motifs (Figure 6b). We found that the sites bound only by TCF7L2 contain the TCF7L2 motif but not the GATA3 motif and the sites bound only by GATA3 contain the GATA3 motif but not the TCF7L2 motif. Interestingly, we found that sites bound by both GATA3 and TCF7L2 are enriched for the GATA3 motif but are not enriched for the TCF7L2 motif. These results suggest that GATA3 may bind to the DNA and recruit TCF7L2 to these sites. To determine if GATA3 can recruit TCF7L2 to a GATA motif in the genome, we introduced small interfering RNAs (siRNAs) specific for GATA3 into MCF7 cells and then tested binding of TCF7L2 to sites bound by both TCF7L2 and GATA3 and to sites bound only by TCF7L2. We found that depletion of GATA3 resulted in the reduction of binding of TCF7L2 at the sites normally bound by both factors but not at the TCF7L2 sites that are not bound by GATA3 (Figure 6c, left panel). In contrast, knockdown of TCF7L2 reduced binding of TCF7L2 but did not reduce binding of GATA3 (Figure 6c, right panel). Thus, GATA3 is necessary for recruiting TCF7L2 to a subset of its genomic binding sites in MCF7 cells but TCF7L2 is not necessary for GATA3 binding to those same sites. We also performed sequential ChIP assays (a TCF7L2 ChIP followed by a GATA3 ChIP and a GATA3 ChIP followed by a TCF7L2 ChIP) to address whether both TCF7L2 and GATA3 are on the same DNA fragments (Additional file 15). In both cases, the sites bound by both TCF7L2 and GATA3 could be enriched by the second antibody, supporting the hypothesis that the two factors are binding at the same time to the same region. To further investigate the hypothesis that GATA3 tethers TCF7L2 to the genome, we sought to determine whether GATA3 interacts with TCF7L2 in MCF7 cell extracts using co-immunoprecipitation. Accordingly, we expressed in MCF7 cells several different FLAG-tagged TCF7L2 constructs that lack either or both of the amino- or carboxy-terminal regions. The amino-terminal region of TCF7L2 mediates the interaction with β-catenin and the carboxy-terminal portion contains the so-called 'E-tail' important for the association with various co-regulators, including CREBBP/EP300 (CREB binding protein/E1A binding protein p300) [32-34]. A predominant isoform lacking the E-tail has been referred to as the B-isoform [17]. Immunoprecipitation of full-length TCF7L2 (the E-isoform) and the B-isoform (which lacks the E-tail) as well as B and E isoforms lacking the amino-terminal β-catenin binding domain (termed EΔ and BΔ) revealed that all isoforms are capable of co-precipitating GATA3 with equal efficiency (Figure 6d). Conversely, immunoprecipitation of GATA3 co-precipitated each of the tested TCF7L2 constructs, albeit with different degrees of efficiency (the full-length E-tail TCF7L2 construct showed the greatest efficiency of co-precipitation with GATA3). Importantly, FLAG immunoprecipitation of extracts prepared from MCF7 cells transfected with an empty vector failed to precipitate GATA3 and control IgG immunoprecipitation reactions failed to precipitate GATA3 and the full-length E construct. Therefore, endogenous GATA3 and exogenously expressed TCF7L2 can interact in MCF7 cells. Taken together, these data show that TCF7L2 and GATA3 interact and co-localize to specific genomic loci in MCF7 cells.

### TCF7L2 functions as a repressor when tethered to the genome by GATA3

**Figure 7:**
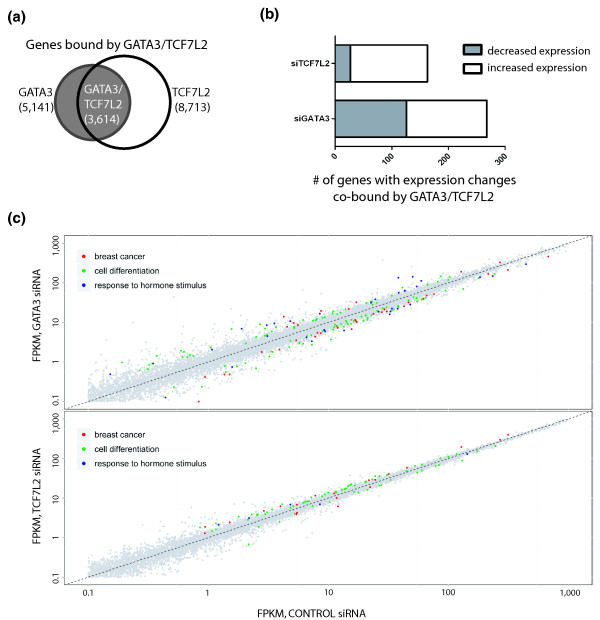
**Transcriptional regulation of TCF7L2 and GATA3 target genes**. **(a) **The nearest gene to each TCF7L2 binding site and the nearest gene to each GATA3 binding site was identified and the two lists were compared to identify 3,614 genes that are potentially regulated by both GATA3 and TCF7L2. **(b) **The expression of the 3,614 GATA3+TCF7L2 bound genes was analyzed in control cells, cells treated with siRNAs to TCF7L2, and cells treated with siRNAs to GATA3; the number of genes whose expression increases or decreases is shown. **(c) **A scatterplot of expression data from RNA-seq experiments. Each point corresponds to one NCBI Reference Sequence (RefSeq) transcript with fragments per kilobase of gene per million reads (FPKM) values for control and siGATA3 or control and siTCF7L2 knockdown samples shown on a log10 scale. The dashed line represents no change in gene expression between the two samples. Differentially expressed genes whose function corresponds to Gene Ontology categories of breast cancer, cell differentiation, and response to hormone stimulus are highlighted.

To establish whether TCF7L2 and GATA3 have a co-regulatory role in the expression of specific target genes, we performed RNA-seq analysis of MCF7 cells before and after knockdown of TCF7L2 or GATA3. We found that the expression of 914 and 469 genes was significantly changed compared to cells treated with control siRNA for GATA3 or TCF7L2, respectively. Many of the genes showing expression changes can be classified as having functions involved in breast cancer, cell differentiation, and response to hormone stimulus (Figure 7c); a list of all genes whose expression was significantly altered by each knockdown can be found in Additional file 16. To identify genes that might be directly co-regulated by GATA3 and TCF7L2, we first identified a set of 3,614 genes that are directly bound by both GATA3 and TCF7L2 (Figure 7a). Then, we analyzed the expression of these 3,614 GATA3+TCF7L2 target genes and found that 268 and 163 genes have significantly altered expression levels in siGATA3- or siTCF7L2-treated cells, respectively (Figure 7b). Approximately half of the set of genes deregulated upon reduction of GATA3 show increased expression and half show decreased expression, suggesting that GATA3 can act as both an activator and a repressor at the GATA3+TCF7L2 target genes. In contrast, most of the genes deregulated by reduction of TCF7L2 show increased expression, suggesting that TCF7L2 functions mainly as a repressor of the set of genes co-bound by TCF7L2 and GATA3. As a final analysis, we identified genes that are co-bound by TCF7L2 and GATA3 and that show expression changes in both the knockdown TCF7L2 cells and in the knockdown GATA3 cells. Although this is a small set of genes, they mainly clustered into two categories. For example, 16 co-bound genes showed an increase in expression in both the TCF7L2 and GATA3 knockdown cells, indicating that both factors were functioning as a repressor of these genes. In addition, we identified ten genes that decreased with GATA3 knockdown but increased upon TCF7L2 knockdown, suggesting that TCF7L2 functioned to negatively modulate GATA3-mediated activation at these genes. A list of the genes that are cooperatively repressed by direct binding of TCF7L2 and GATA3 and a list of genes for which recruitment of TCF7L2 antagonizes GATA3-mediated activation are shown in Table 4.

**Table 4:** Genes repressed by TCF7L2 via a GATA motif

Cooperative repression by TCF7L2 and GATA3	TCF7L2 antagonizes GATA3-mediated activation
ABCA12	CCND1
APP	COL5A1
CAPN2	CYP24A1
CDH11	GPRC5A
EPHA4	KRT80
GALC	LYPD1
LTBP1	MAP2
LYPD3	RASGRP1
MARCKS	RHOBTB3
NCOA3	TGFB2
NFAT5	TGFBR2
PAM	
RNF145	
S100A10	
SECISBP2L	
SORL1	

## Discussion

The TCF7L2 transcription factor has been linked to a variety of human diseases such as type 2 diabetes and cancer [3,7-9,35]. To investigate the mechanisms by which this site-specific DNA binding transcriptional regulator can impact on such diverse diseases, we performed ChIP-seq analysis for TCF7L2 in 6 different human cell lines, identifying more than 116,000 non-redundant binding sites, with only 1,864 sites being common to all 6 cell types. Several striking discoveries that came from our ChIP-seq analysis of the 6 different cell lines are: i) TCF7L2 has multiple binding sites near each target gene; ii) TCF7L2 has developed cell type-specific mechanisms for regulating a set of approximately 14,000 genes; iii) TCF7L2 binds to more than 40% of the active enhancers in each of the 6 cancer cell lines; and iv) TCF7L2 functions as repressor when recruited to the genome via tethering by the master regulator GATA3.

By analysis of the TCF7L2 ChIP-seq datasets from 6 different human cancer cell lines, we identified 116,270 TCF7L2 binding sites, with each cell type having approximately 25,000 to 50,000 TCF7L2 peaks. We note that another group has examined TCF7L2 binding in human HCT116 cells [12], identifying only 1,095 binding sites. It is not clear why Zhao and colleagues [12] identified such smaller numbers of TCF7L2 binding sites in HCT116 cells, but it is not likely due to the antibody specificity (the antibodies used in both studies give similar patterns on western blots). It is more likely that the 30-fold difference in peak number is due to the ChIP protocol. Zhao *et al*. [12] used protein A agarose beads, whereas we used magnetic protein A/G beads; we have found that protein A agarose beads produce low signals in many ChIP assays (unpublished data). Interestingly, the 116,270 TCF7L2 binding sites that we identified correspond to only 14,193 genes, with each target gene having an average of 8.2 TCF7L2 binding sites. Many of these binding sites are cell type-specific, as exemplified by the fact that there are only three to four TCF7L2 binding sites per target gene in any one cell type (Figure 2c).

Cell type-specific binding patterns suggest that TCF7L2 binds cooperatively to the genome along with cell type-specific factors. For example, the AP1 (activator protein 1) motif is enriched in the sets of HCT116-specific and MCF7-specific TCF7L2 binding sites. Interestingly, TCF7L2 has previously been shown to physically interact with JUN (which is one of the heterodimeric components of AP1) and it has been suggested that the JUN and TCF7L2 interaction is a molecular mechanism that integrates the activation of the TCF and CTNNB1 pathway by the JNK (Jun N-terminal kinase) pathway [36]. Although ChIP-seq data for AP1 components is not available for HCT116 or MCF7 cells, there are 7,400 genomic locations that are bound by TCF7L2 in HCT116 cells that are also bound by JUN in HeLa cells [11]; it is likely that a much larger number of co-localizing regions would be identified if the datasets were from the same cell type. Our detailed bioinformatic analysis of the HepG2-specific TCF7L2 peaks suggested that HNF4α and FOXA2 might be binding partners of TCF7L2 in this cell type. A previous study had shown that FOXA2 and HNF4α colocalize at a subset of sites in mouse liver [37], but that study did not examine the relationship of these sites with TCF7L2 binding. Therefore, we experimentally validated our bioinformatic prediction by comparing ChIP-seq data for all three factors. We found that greater than 50% of the TCF7L2 HepG2-specific binding sites are also bound by the liver transcription factors HNF4α and FOXA2, suggesting that this trio of factors cooperate in gene regulation. Based on the identification of motifs for all three factors in the TCF7L2 peaks, we suggest that TCF7L2, HNF4α, and FOXA2 all bind directly to the DNA, perhaps with the liver-specific factors helping to stabilize TCF7L2 genomic binding to particular enhancer regions in HepG2 cells. HNF4α and FOXA2 have been shown to be critical determinants of hepatocyte identity; Hnf4α plus Foxa1, Foxa2, or Foxa3 can convert mouse embryonic and adult fibroblasts into cells that closely resemble hepatocytes *in vitro *[38]. The induced hepatocyte-like cells had multiple hepatocyte-specific features and reconstituted damaged hepatic tissues after transplantation. Future studies should address a potential role of TCF7L2 in hepatocyte identity.

**Figure 8:**
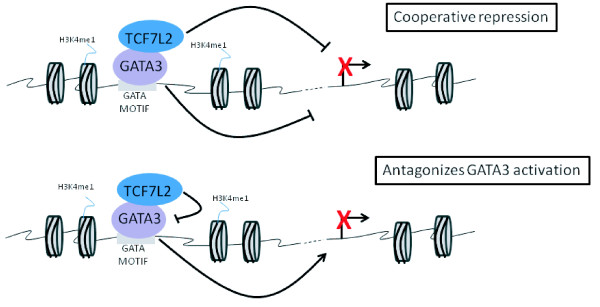
**Two modes of TCF7L2-mediated transcriptional repression of GATA3 target genes**. **(a) **GATA3 tethers TCF7L2 to the genome and both factors cooperate to repress target genes. **(b) **GATA3 tethers TCF7L2 to the genome with TCF7L2 antagonizing GATA3-mediated transcriptional activation.

Bioinformatic analysis of the MCF7-specific TCF7L2 sites revealed that the GATA3 motif was highly enriched and experimental analysis of MCF7 GATA3 ChIP-seq data showed that nearly one-half of the MCF7-specific TCF7L2 binding sites co-localize with GATA3. Interestingly, we found that the TCF7L2 motif was not enriched in the regions bound by both TCF7L2 and GATA3. These results suggested that perhaps GATA3 binds directly to the DNA at these sites and tethers TCF7L2 to the genome at the MCF7-specific TCF7L2 binding sites Accordingly, we showed that depletion of GATA3 reduced recruitment of TCF7L2 to a subset of genomic sites. We also demonstrated that TCF7L2 functions mainly as a repressor when tethered to the genome via GATA3. At some genes, TCF7L2 cooperatively represses genes with GATA3 but at other genes TCF7L2 antagonizes GATA3-mediated activation (Figure 8).

Specification of cell phenotypes is achieved by sets of master transcriptional regulators that activate the genes specific for one cell fate while repressing genes that specify other cell fates. The GATA factors, which include six site-specific DNA binding proteins that bind to the sequence (A/T)GATA(A/G), are master regulators that govern cell differentiation [39-44]. For example, GATA1-3 have been linked to the specification of different hematopoietic cell fates and GATA4-6 are involved in differentiation of cardiac and lung tissues. Also, GATA3 is the most highly enriched transcription factor in the mammary epithelium, has been shown to be necessary for mammary cell differentiation, and is specifically required to maintain the luminal cell fate [43,44]. Studies of human breast cancers have shown that GATA3 is expressed in early stage, well-differentiated tumors but not in advanced invasive cancers. In addition, GATA3 expression is correlated with longer disease-free survival and evidence suggests that it can prevent or reverse the epithelial to mesenchymal transition that is characteristic of cancer metastasis [45]. Our studies show that TCF7L2 cooperates with the master regulator GATA3 to repress transcription in the well-differentiated MCF7 breast cancer cell line and suggest that a TCF7L2-GATA3 complex may be a critical regulator of breast cell differentiation.

Our finding that TCF7L2 co-localizes and cooperates in gene regulation with a GATA factor in MCF7 breast cancer cells is similar to a recent study of TCF7L2 in hematopoietic cells. Trompouki *et al*. [13] showed that in hematopoietic cells, TCF7L2 co-occupies sites with GATA1 and GATA2, which are master regulators of blood cell differentiation. Both the TCF7L2 motif and the GATA motif were found at the co-bound sites (suggesting adjacent binding of the two factors, not tethering) and TCF7L2 functioned as a transcriptional activator at those sites. In contrast, our studies indicate that co-localization of TCF7L2 with GATA3 in MCF7 cells is not due to adjacent binding but rather TCF7L2 is tethered to the genome by interaction with GATA3 binding to a GATA motif and that this tethering results in transcriptional repression. A study of *Drosophila *TCF binding to the *Ugt36Bc *upstream region indicated that TCF represses transcription of the *Ugt36Bc *gene by binding to non-traditional TCF motifs [46]. Interestingly, the three *Ugt36Bc *TCF sites (AGAAAT, AGATAA, AGATAA) are almost identical to the GATA3 motif. Blauwkamp *et al*. [46] suggest that the sequence to which TCF binds has an important function in determining whether a gene will be activated or repressed. Their studies did not address whether TCF bound directly to the GATA-like motifs. However, based on our studies, it would be worthwhile to investigate a possible genomic tethering mechanism of TCF by GATA factors in *Drosophila*.

## Conclusions

Our studies reveal numerous new insights into TCF7L2-mediated gene regulation and suggest that TCF7L2 cooperates with other site-specific DNA binding factors to regulate transcription in a cell type-specific manner. Specifically, we show that TCF7L2 has highly cell type-specific binding patterns, co-localizes with different factors in different cell types, and can be tethered to the DNA by GATA3 in breast cancer cells. Our work, in combination with other studies [13,47], suggests that TCF7L2 may play a critical role in creating and maintaining differentiated phenotypes by cooperating with cell type-specific master regulators such as HNF4α and FOXA2 in liver cells and GATA3 in breast cells. Both FOXA and GATA family members have been classified as pioneer factors, that is, transcription factors that can access their binding sites when other factors cannot, helping to create open chromatin to enable subsequent binding of other factors [48]. It is possible that FOXA2 and GATA3 serve as pioneer factors that enhance the ability of TCF7L2 to access its sites in liver and breast cells. In addition to having cell type-specific partners, there are many different isoforms of TCF7L2. Although the major isoforms of TCF7L2 are similar in most cell types, it is possible that minor isoforms contribute to the cell type-specificity of TCF7L2 binding via interaction of co-localizing proteins with alternatively encoded exons of TCF7L2. We anticipate that future studies employing isoform-specific antibodies to identify TCF7L2 binding sites in normal and diseased tissues will provide additional insight into the transcriptional networks that are altered in diseases such as type 2 diabetes, pancreatic cancer, and coronary artery disease.

## Materials and methods

### Cell culture

The human cell lines HCT116 (ATCC #CCL-247), HepG2 (ATCC # HB-8065), HEK293 (ATCC #CRL-1573), MCF7 (ATCC #HTB-22), HeLa (ATCC #CCL-2.) and PANC1 (ATCC #CRL-1469) were obtained from the American Type Culture Collection. HCT116 cells were grown in McCoy's 5A Medium supplemented with 10% fetal bovine serum and 1% penicillin/streptomycin until 80% confluent, whereas HepG2, HEK293, MCF7, HeLa and PANC1 cells were grown in Dulbecco's modified Eagle's medium supplemented with 10% fetal bovine serum, 2 mM L-glutamine and 1% penicillin/streptomycin) until 75 to 90% confluent.

### siRNA-mediated knockdown

All siRNAs were purchased from Dharmacon (Thermo Fisher Scientific-Dharmacon Products, Lafayette, CO, USA; ON-TARGET plus SMART pool - Human GATA3, TCF7L2 and Non-Targeting siRNA) and transfected using Lipofectamine™ 2000 Transfection Reagent according to the manufacturer's instructions (Life Technologies, Grand Island, NY, USA). Then, 48 to 56 h following transfection, cells were either crosslinked for ChIP assays or collected for RNA and protein extraction.

### ChIP-seq assays

The antibodies used for ChIP-seq were: TCF7L2 (Cell Signaling Technology, Danvers, MA, USA; C48H11 #2569), GATA3 (Santa Cruz Biotechnology, Santa Cruz, CA, USA; #sc-268), H3K4me1 (Cell Signaling Technology, Danvers, MA, USA; 9723S lot1), and H3K27Ac (Abcam, Cambridge, MA, USA; Ab4729 lot #GR16377-1). The TCF7L2 antibody will detect both major isoforms of TCF7L2. See Additional file 2 for details of all ChIP-seq experiments. For all factor or histone modification and cell type combination, we performed duplicate ChIP-seq experiments using chromatin from two different cell culture dates. For the TCF7L2 ChIP-seq assays, 500 μg chromatin was incubated with 25 μg of antibody; for the GATA3 experiments, 600 μg chromatin was incubated with 50 μg of antibody; and for the histone ChIP-seq experiments, 10 to 12 μg chromatin and 8 to 10 μg of antibody were used. TCF7L2 and histone ChIP assays were performed as described previously [49] using protein A/G magnetic beads to collect the immunoprecipitates. GATA3 ChIP-seq experiments were performed using StaphA (Sigma-Aldrich, St. Louis, MO, USA) to collect the immunoprecipitates [50]. After qPCR confirmed enrichment of target sequences in ChIP versus input samples, libraries were created as previously described with minor modifications [49]. Gel size selection of the 200 to 500 bp fraction (TCF7L2 and histones) or the 300 to 600 bp fraction (GATA3) was conducted after the adapter ligation step, followed by 15 amplification cycles. qPCR (see Additional file 17 for a list of primers used in this study) was performed to confirm enrichment of targets in the libraries and then the libraries were analyzed using an Illumina GAIIx. Sequence reads were aligned to the UCSC human genome assembly HG19 using the Eland pipeline (Illumina).

### ChIP-seq data processing

The BELT program [24] and Sole-search [11,51] were used to identify peaks for TCF7L2 and for modified histones. We used the ENCODE overlap rules to evaluate the reproducibility of the two biological replicates for each factor or histone modification and cell-type combination. For this, we first truncated the peak lists of the two replicates for a given factor/cell-type combination so that both the A and B replicate peak list were the same length. Then, we overlapped the top 40% of the replicate A peak list with the entire replicate B peak list (and vice versa). ENCODE standards state that approximately 80% of the top 40% set should be contained in the larger set. After determining that replicate datasets met this standard (Additional file 4), we merged the two replicates and called peaks on the merged dataset. To determine if we had identified the majority of the TCF7L2 peaks in each cell type, we performed a saturation analysis. We randomly selected different percentages of the reads (10%, 20%, 30%,...,100%) from the merged datasets from the TCF7L2 ChIP-seq experiments for each cell line and called peaks using the BELT program; each merged dataset was analyzed three times. The number of peaks identified in each subset of the total reads was plotted to demonstrate that we had enough reads for each dataset to identify the majority of peaks (Additional file 7).

### RNA-seq

RNA was extracted using Trizol Reagent (Life Technologies) following the suggested protocol; 2 μg of each RNA sample was used with the Illumina TruSeq RNA Sample Prep Kit (catalogue number RS-122-2001) to make RNA libraries following the Illumina TruSeq RNA Sample preparation Low-Throughput protocol. Briefly, RNA was fragmented, then first-strand cDNA was prepared using the kit-supplied 1st Strand Master Mix and user-supplied Superscript III (Life Technologies, catalogue number 18080-051) followed by second strand cDNA synthesis. The Illumina protocol and reagents were used to complete the library preparation, with 12 cycles of PCR amplification. Libraries were sequenced using an Illumina GAIIx and analyzed as described in Additional file 3.

### ChIP-qPCR assays

ChIP assays were performed as described in the ChIP-seq section, except that 30 μg equivalents of DNA was used for each ChIP reaction. The ChIP eluates were analyzed by qPCR using the Bio-Rad SsoFast™ EvaGreen^®^ Supermix (catalogue number 172-5202) according to the manufacturer's instructions (Bio-Rad, Hercules, CA, USA).

### Generation of TCF7L2 expression constructs and co-immunoprecipitation assays

TCF7L2 expression constructs were generated by PCR amplification of cDNA prepared from RNA isolated from MCF7 cell cultures and used for GATEWAY cloning into the pTRED-N-FLAG expression vector, which contains an amino-terminal FLAG tag. Control empty vector or an expression construct was transfected into MCF7 cells using Lipofectamine™ 2000 according to the manufacturer's instructions (Life Technologies); 36 h following transfection, cells were harvested and lysed in ice-cold NP-40 lysis buffer (phosphate-buffered saline, 0.25% NP-40, 0.1% sodium-deoxycholate, 2 mM phenylmethylsulfonyl fluoride (PMSF) and 10 μg/ml leupeptin and aprotinin) for co-immunoprecipitation assays. Following extraction on ice for 30 minutes and clarification by centrifugation, soluble protein extracts were diluted 1:10 with lysis buffer and incubated with either an anti-FLAG M2 agarose conjugated antibody (Sigma catalogue number A2220), an anti-GATA3 conjugated antibody (Santa Cruz HG3-31-AC), or a control rabbit IgG agarose conjugated antibody (Sigma catalogue number A2909) for 4 hours at 4°C. The beads were then washed four times and eluted with SDS-PAGE sample buffer prior to SDS-PAGE and western blot analysis using antibodies specific for GATA3 (Santa Cruz HG3-31) or FLAG (Sigma catalogue number A8592).

### Data access

All data are publicly available via the UCSC Genome Preview Browser and/or has been submitted to the Gene Expression Omnibus (information concerning how to access the data is provided in Additional file 2).

## Abbreviations

AP1: activator protein 1; bp: base pair; ChIP: chromatin immunoprecipitation; CTNNB1: catenin beta 1; FOX: forkhead box; GATA: GATA binding protein; H3K27Ac histone H3 acetylated on lysine 27; H3K4me1: histone H3 monomethylated on lysine 4; HNF: hepatocyte nuclear factor; PCR: polymerase chain reaction; qPCR: quantitative PCR; siRNA: small interfering RNA; TCF7L2: transcription factor 7-like 2.

## Competing interests

The authors declare that they have no competing interests.

## Authors' contributions

All authors have read and approved the manuscript for publication. SF performed ChIP-seq assays and bioinformatics analyses, participated in the study design and coordination, and helped to draft the manuscript. RW performed bioinformatic analyses. YGT, LY, MG, and HW performed ChIP-seq assays and participated in analysis of the data. VXJ participated in the design of the study, performed data analyses, and helped to draft the manuscript. PJF conceived of the study, participated in its design and coordination, performed data analyses, and drafted the manuscript.

## Supplementary Material

Additional file 1 **Figure S1 - antibody validation**. Click here for file

Additional file 2 **Table S1 - summary of ChIP-seq and RNA-seq experiments**. All ChIP-seq experiments performed by the Farnham laboratory are listed; GEO numbers are provided and the data are availableat the UCSC genome browser Click here for file

Additional file 3 **Supplementary Methods**. Click here for file

Additional file 4 **Table S2 - ChIP-seq reproducibility**. To determine the reproducibility of the ChIP-seq data, we used the method of evaluating replicates as described in the ENCODE Standards document [53]. Briefly, the ENCODE consortium rules are as follows: '80% of the top 40% of the targets identified in one replicate should be contained within the list of targets from the other replicate'. This metric was chosen based on experiences of all the ENCODE production groups to allow an achievable threshold of reproducibility while producing high quality target lists. All ChIP-seq data for site-specific factors submitted to the UCSC browser as part of ENCODE have to pass this quality metric and, as can be seen in Table S2, all of the TCF7L2 data in our manuscript have passed. We note that the metric for reproducibility of 'broad peak' histone marks (such as H3K4me1) has not yet been established by ENCODE. Due to difficulties in calling peaks for such histone marks, the overlap is sometimes lower than 80%. Click here for file

Additional file 5 **Table S3 - all TCF7L2 peaks in six cell types**. TCF7L2 peaks were called using BELT [54] and the merged datasets for each cell type (see Additional file 4 for the peak calling parameters for each dataset). Click here for file

Additional file 6 **Table S7 - summary of TCF7L2 peak characteristics**. TCF7L2 peaks were called using BELT [54] and the merged datasets for each cell type (see Additional file 4 for the peak calling parameters for each dataset). Click here for file

Additional file 7 **Figure S2 - saturation analysis**. Click here for file

Additional file 8 **Table S9 - amplified regions in the six cancer cell lines**. Sole-search was used to call peaks for TCF7L2 in each of the six cancer cell lines, using input from each line as the specific control. One novel feature of Sole-search is that it provides a list of all amplified regions found in the input control. For each cancer cell line, a worksheet of all amplified regions is provided; column F indicates the fold amplification and column I indicates the chromosomal coordinates of the amplified region Click here for file

Additional file 9 **Table S10 - TCF7L2 peaks in amplified regions**. The TCF7L2 peaks from each cell line were overlapped with the amplified regions from that same cell line. Presented in this table is a summary of all the overlaps and a worksheet for each cell line that lists each peak that is found in the amplified regions (column A indicates the chromosome and columns D and E indicate the chromosomal coordinates of the amplified region that contains the peak; column F indicates the fold amplification of the amplified region; and column I indicates the chromosomal coordinate of the TCF7L2 peak and the height of the peak). Click here for file

Additional file 10 **Table S4 - TCF7L2 peaks unique to each of the six cell types**. To identify cell type-specific TCF7L2 peaks for a particular cell, we first combined the five sets of peaks from the other cell types, and then identified the unique set of peaks for the given cell type by removing sites in common with the combined set. For these analyses, the merged replicate TCF7L2 datasets were used. Click here for file

Additional file 11 **Figure S3 - ChIP-qPCR validation of TCF7L2 sites**. Click here for file

Additional file 12 **Figure S4 - TCF7L2 binds to cell type-specific enhancer regions**. Click here for file

Additional file 13 **Table S6 - TCF7L2 binding motifs in six cell types**. We used our ChIPMotifs program to identify two canonical TCF7L2 motifs, W1 of 6 bp and W2 of 8 bp, for each cell type. We then used each of two motifs' position weight matrices to scan the sequences of the peaks to determine how many peaks contained the motifs; we examined the set of all peaks and the set of cell type-specific peaks for all six cell types. Click here for file

Additional file 14 **Figure S5 - motif recovery percentage plots**. Click here for file

Additional file 15 **Figure S6 - Re-ChIP analysis of GATA3 and TCF7L2 sites**. Click here for file

Additional file 16 **Table S8 - RNA-seq analysis of TCF7L2 and GATA3 knockdowns**. The RNAseq data were processed by TopHat and Cufflinks programs essentially as described [55]. Click here for file

Additional file 17 **Table S5 - primers used in ChIP-qPCR analyses**. The sequences of positive and negative control primers used for ChIP-qPCR. Click here for file
