# Supplementary material for: Cell type-specific binding patterns reveal that TCF7L2 can be tethered to the genome by association with GATA3
Source: Genome Biol. 2012 Sep 5;13(9):R52. doi: 10.1186/gb-2012-13-9-r52 (PMC3491396; doi:10.1186/gb-2012-13-9-r52)
Supplement: Additional file 1 — Figure S1 - antibody validation. [file gb-2012-13-9-r52-S1.pdf]

Figure S1

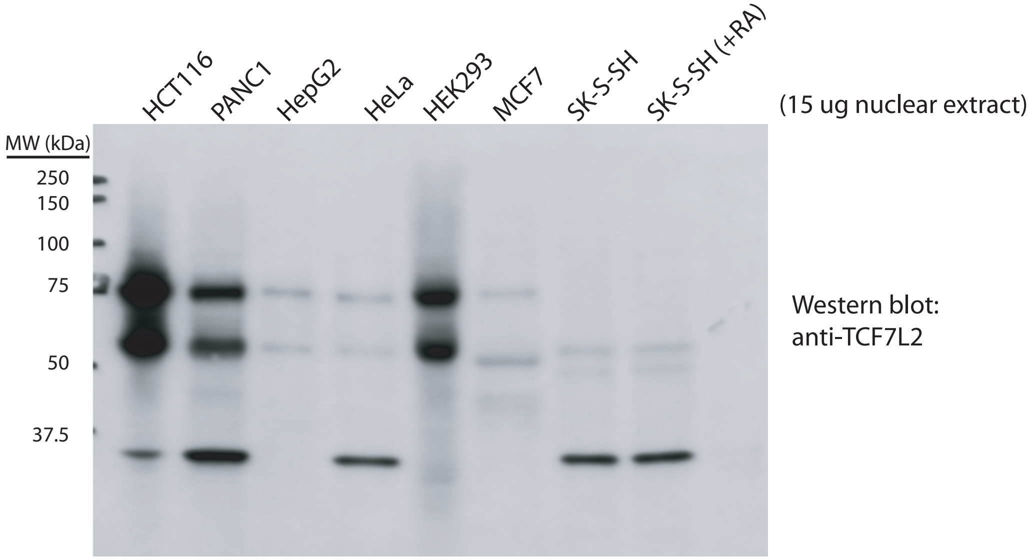

Figure S1. Western blot for TCF7L2 in several human cell lines. Alternative splicing produces two major forms of TCF7L2 in most cells, a cluster of isoforms of ~79 kD and a cluster of isoforms of ~58 kD. We also note that there is an additional ~35 kD isoform in HCT116, PANC1, and HeLa cells. Although others (Vacik et al. 2011) have characterized a ~35 kD TCF7L2 isoform that lacks exons 1-4, the small isoform shown here must contain the amino terminal exons because it is recognized by antibodies against the N terminus of the TCF7L2. The DNA binding domain is encoded by exons 10 and 11 so perhaps the 35 kD isoform does not have DNA binding ability and thus would not contribute to the ChIP-seq data; if so, this would be similar to a 20 kD TCF7L2 isoform that is produced via an alternative polyadenylation signal in intron 4 (Locke et al. 2011).
