# Supplementary material for: Cell type-specific binding patterns reveal that TCF7L2 can be tethered to the genome by association with GATA3
Source: Genome Biol. 2012 Sep 5;13(9):R52. doi: 10.1186/gb-2012-13-9-r52 (PMC3491396; doi:10.1186/gb-2012-13-9-r52)
Supplement: Additional file 3 — Supplementary Methods. [file gb-2012-13-9-r52-S3.pdf]

Additional file 3: Supplementary Methods

Identification of TCF7L2 binding sites: We applied our peak-calling detection program BELT (http://motif.bmi.ohio-state.edu/W-ChIPeaks/) to identify the TCF7L2 binding site for each replicate ChIP-seq dataset using a range of parameters, e.g. a bin size of 100-500 bp and thresholds of 0.90-0.99. We then chose the optimal parameters that produced an FDR less than 10% (most have FDRs less than 5%); the parameters for each called peak set are indicated in Supplementary Figure S2. We compared the peak sets of the replicates, using the ENCODE overlap rules to determine reproducibly. ENCODE rules are that Rep A and B peak lists are truncated to the same length, followed by comparing top 40% of the replicate A peak list with the entire replicate B peak list (and vice versa). If ~80% of the top 40% set is contained in the larger set, then the data is considered to be very reproducible. After determining that our replicate datasets met this standard (see **Supplementary Table S2**), we merged the reads from the two replicates and called peaks on the merged dataset.

We used a saturation analysis strategy to determine a final set of TCF7L2 peaks for each cell type. We randomly selected ten different subsets of reads, with each subset corresponding to different percentages of the reads (10%, 20%, 30%,.., 100%) from the merged TCF7L2 datasets for each cell type and called peaks on each subset using the BELT program. We used two parameters T, (enrichment threshold) and FDR, from calling the peaks in the subset of 100% reads as reference parameters, to call peaks for other subsets having the lower percentages of reads. This ensured that the quality and quantity of peaks were comparable among the different subsets; each subset was analyzed three times. We then applied Pearson Correlation Coefficient (PCC) to determine statistical correlations among the number of peaks identified from three randomized subsets (pairwise PCC > 0.99). Finally, the number of peaks identified in each subset was plotted to demonstrate that we had enough reads for each dataset to identify the majority of peaks (**Supplementary Figure S2**). After determining a final set of peaks for each cell type, we computed the average and median of scores and width of peaks for each set (**Supplementary Table S11**).

Identification of cell-type specific TCF7L2 binding sites: To identify cell type-specific TCF7L2 peaks for a particular cell, we first combined the five sets of peaks from the other cell types, then we identified the unique set of peaks for the given cell type by removing sites in common with the combined set. After determining a final set of each cell type-specific peaks, we computed the average and median of scores and width of peaks for each set (**Supplementary Table S11**).

Identification of a combined set of TCF7L2 binding sites: We started by combining two sets of peaks from any of two cell types to produce a single, non-redundant set of peaks. We then compared this 2-way merged set to the set of TCF7L2 peaks from the 3rd cell type, and identified a new set of unique peaks in the 3rd dataset. We then added the newly identified unique set of peaks to 2-way merged set, producing a 3-way merged set of unique peaks. We kept doing this until we had incorporated all six cell types, thus identified a final set of combined peaks.

Analysis of tag density and generation of heatmaps
Matrices for the tag density heatmaps were generated using HOMER (Heinz et al. *Molecular Cell.* 2010;38:576-589), clustered in Cluster3 (de Hoon et al. *Bioinformatics (Oxford, England).* 2004;20:1453-1454) and visualized in TreeView (Saldanha AJ. *Bioinformatics (Oxford, England).* 2004;20:3246-3248). The TCF7L2 tag density heatmap from Figure 1 was created by sorting TCF7L2 unique peaks by rank and combining the top 500 sites for each cell-line to a single bed file to create a matrix file which was clustered by Euclidean distance. H3K27ac and

H3K4me1 as well as HNF4 and FoxA2 tag density heatmaps from Figure 3B and Figure 5C were created using k-means clustering (k=3) using Cluster3.

Motif analysis

Motif analysis was performed on TCF7L2 sequences positioned on the center of the binding site with HOMER using the default settings. The motif density profile relative to the center of the binding site was generated using the HOMER annotatePeaks tool with bins of 10 bp searching for the identified motif matrix files.

RNAseq analysis

The RNAseq data was processed by TopHat and Cufflinks programs essentially as described (Trapnell et al. *Nat Protoc.* 2012;7:562-78). The genes containing a TCF7L2 or GATA3 peak were identified using the Solesearch location analysis tool (Blahnik et al. *Nucleic Acids Res* 2010, 38:e13). The ontology terms were obtained using DAVID gene-annotation enrichment analysis (Huang et al. Nature Protoc. 2009;4:44-57; Huang et al. Nucleic Acids Res. 2009;37:1-13).
