# Supplementary material for: Cell type-specific binding patterns reveal that TCF7L2 can be tethered to the genome by association with GATA3
Source: Genome Biol. 2012 Sep 5;13(9):R52. doi: 10.1186/gb-2012-13-9-r52 (PMC3491396; doi:10.1186/gb-2012-13-9-r52)
Supplement: Additional file 4 — Table S2 - ChIP-seq reproducibility. To determine the reproducibility of the ChIP-seq data, we used the method of evaluating replicates as described in the ENCODE Standards document [53]. Briefly, the ENCODE consortium rules are as follows: '80% of the top 40% of the targets identified in one replicate should be contained within the list of targets from the other replicate'. This metric was chosen based on experiences of all the ENCODE production groups to allow an achievable threshold of reproducibility while producing high quality target lists. All ChIP-seq data for site-specific factors submitted to the UCSC browser as part of ENCODE have to pass this quality metric and, as can be seen in Table S2, all of the TCF7L2 data in our manuscript have passed. We note that the metric for reproducibility of 'broad peak' histone marks (such as H3K4me1) has not yet been established by ENCODE. Due to difficulties in calling peaks for such histone marks, the overlap is sometimes lower than 80%. [file gb-2012-13-9-r52-S4.pdf]

Cell-line	Overlap	Peaks	40% Overlap Rule
HCT116	TCF7L2 rep1 vs rep2	40,436	97%
HCT116	TCF7L2 rep2 vs rep1	25,707	98%
HCT116	H3K4me1 rep1 vs rep2	34,937	98%
HCT116	H3K4me1 rep2 vs rep1	55,192	98%
HCT116	H3K27Ac rep1 vs rep2	29,756	97%
HCT116	H3K27Ac rep2 vs rep1	28,077	94%
HEK293	TCF7L2 rep1 vs rep2	10,743	90%
HEK293	TCF7L2 rep2 vs rep1	19,999	89%
HepG2	TCF7L2 rep1 vs rep2	9,323	88%
HepG2	TCF7L2 rep2 vs rep1	47,019	87%
HepG2	H3K4me1 rep1 vs rep2	94,830	100%
HepG2	H3K4me1 rep2 vs rep1	88,688	100%
HepG2	H3K27Ac rep1 vs rep2	25,192	100%
HepG2	H3K27Ac rep2 vs rep1	25,325	100%
Hela	TCF7L2 rep1 vs rep2	28,652	69%
Hela	TCF7L2 rep2 vs rep1	51,314	90%
HeLa	H3K4me1 rep1 vs rep2	58,404	99%
HeLa	H3K4me1 rep2 vs rep1	61,672	98%
HeLa	H3K27Ac rep1 vs rep2	27,582	100%
HeLa	H3K27Ac rep2 vs rep1	29,505	100%
PANC1	TCF7L2 rep1 vs rep2	8,600	99%
PANC1	TCF7L2 rep2 vs rep1	14,201	99%
PANC1	H3K4me1 rep1 vs rep2	29,669	100%
PANC1	H3K4me1 rep2 vs rep1	60,066	100%
PANC1	H3K27Ac rep1 vs rep2	42,534	98%
PANC1	H3K27Ac rep2 vs rep1	28,643	92%
MCF7	TCF7L2 rep1 vs rep2	23,300	98%
MCF7	TCF7L2 rep2 vs rep1	16,349	91%
MCF7	GATA3 rep1 vs rep2	14,504	96%
MCF7	GATA3 rep2 vs rep1	10,179	97%
MCF7	H3K4me1 rep1 vs rep2	21,276	71%
MCF7	H3K4me1 rep2 vs rep1	6,536	76%
MCF7	H3K27Ac rep1 vs rep2	26,049	99%
MCF7	H3K27Ac rep2 vs rep1	23,369	100%
