# Supplementary material for: Cell type-specific binding patterns reveal that TCF7L2 can be tethered to the genome by association with GATA3
Source: Genome Biol. 2012 Sep 5;13(9):R52. doi: 10.1186/gb-2012-13-9-r52 (PMC3491396; doi:10.1186/gb-2012-13-9-r52)
Supplement: Additional file 6 — Table S7 - summary of TCF7L2 peak characteristics. TCF7L2 peaks were called using BELT [54] and the merged datasets for each cell type (see Additional file 4 for the peak calling parameters for each dataset). [file gb-2012-13-9-r52-S6.pdf]

Supplementary Table S7: Summary of Peak Characteristics

Cell type	Peak width range	Peak width median	Peak width average	Peak score range	Peak Score median	Peak score average
HepG2	299-4949	599	698	3.82-8.72	4.62	4.88
HCT116	199-4799	799	792	3.50-8.91	4.38	4.59
HEK293	249-4999	749	828	3.59-9.11	4.01	4.22
HeLa	299-4799	599	638	4.20-9.42	4.81	5.04
MCF7	399-4999	599	714	3.50-9.13	4.00	4.21
PANC1	199-4999	599	774	3.20-8.67	3.75	4.01

All peaks

Cell type	Peak width range	Peak width median	Peak width average	Peak score range	Peak Score median	Peak score average
HepG2	299-4199	599	655	3.82-8.34	4.57	4.79
HCT116	199-2799	599	603	3.50-8.15	3.96	4.17
HEK293	249-4999	749	780	3.59-7.77	3.91	4.10
HeLa	299-3749	449	486	4.20-8.14	4.53	4.70
MCF7	399-4999	599	704	3.50-8.17	3.85	4.05
PANC1	199-4999	599	645	3.20-7.31	3.50	3.73

Cell type- specific peaks
