# Supplementary material for: Cell type-specific binding patterns reveal that TCF7L2 can be tethered to the genome by association with GATA3
Source: Genome Biol. 2012 Sep 5;13(9):R52. doi: 10.1186/gb-2012-13-9-r52 (PMC3491396; doi:10.1186/gb-2012-13-9-r52)
Supplement: Additional file 7 — Figure S2 - saturation analysis. [file gb-2012-13-9-r52-S7.pdf]

Supplementary Figure S2: Saturation analysis plots. We randomly selected ten different subsets of reads corresponding to different percentages of the total reads (10%, 20%, 30%,.., 100%) for each cell type from the merged TCF7L2 ChIP-seq datasets and called peaks on each subset using the BELT program. These analyses showed that for each cell type the peak numbers reach saturation when the peaks are identified from subsets of peaks above 80% of the total reads for that cell type.

Saturation analysis to determine a final set for HepG2 (0.95/150)

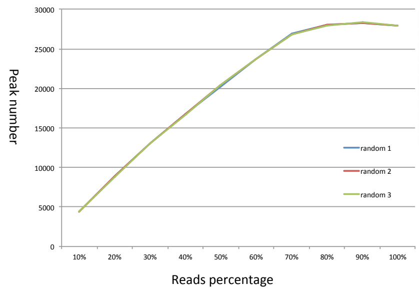

	random 1	random 2	random [illegible]
10%	4421	4360	[illegible]
20%	8884	8872	[illegible]
30%	13001	12980	[illegible]
40%	16779	16808	[illegible]
50%	20236	20378	[illegible]
60%	23705	23710	[illegible]
70%	26983	26860	[illegible]
80%	27988	28029	[illegible]
90%	28274	28244	[illegible]
100%	27912	27912	[illegible]

Saturation analysis to determine a final set for HCT116 (0.94/200)

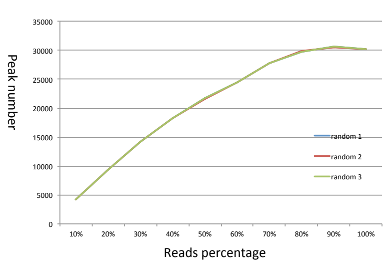

	random 1	random 2	random 3
10	4151	4129	4162
20	9292	9285	9281
30	14208	14131	14122
40	18329	18312	18295
50	21725	21667	21675
60	24448	24487	24423
70	27763	27774	27766
80	29728	29890	29805
90	30627	30532	30624
100	30266	30266	30266

Saturation analysis to determine a final set for HEK293 (0.93/250)

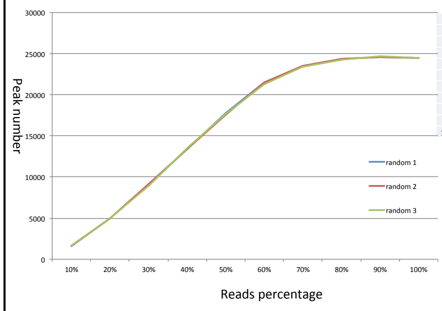

	random 1	random 2	random 3
10	1594	1649	1621
20	4987	4974	4999
30	9104	9180	9016
40	13460	13458	13527
50	17756	17606	17728
60	21460	21551	21307
70	23407	23520	23431
80	24389	24329	24243
90	24601	24539	24686
100	24457	24457	24457

Saturation analysis to determine a final set for PANC1(0.91/200)

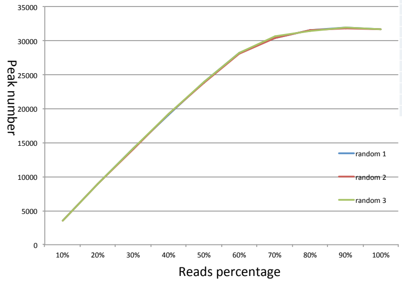

	random 1	random 2	random 3
10	3543	3499	3517
20	8916	8887	8908
30	14152	14126	14176
40	19106	19187	19271
50	23953	23895	23968
60	28188	28117	28194
70	30475	30455	30654
80	31627	31635	31487
90	31977	31861	31922
100	31744	31744	31744

Saturation analysis to determine a final set for MCF7(0.92/200)

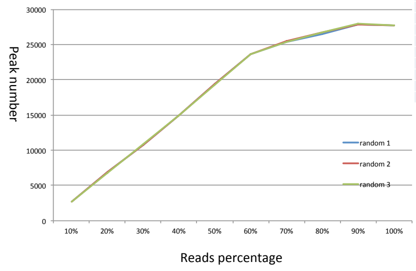

	random 1	random 2	random 3
10	2669	2695	2679
20	6895	6924	6844
30	10750	10782	10869
40	14966	14999	14936
50	19470	19382	19357
60	23656	23647	23581
70	25332	25540	25418
80	26529	26744	26692
90	27906	27818	27970
100	27721	27721	27721

Saturation analysis to determine a final set for HeLa (0.9/150)

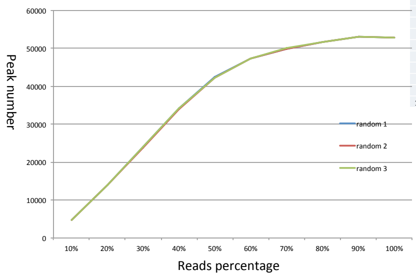

	random 1	random 2	random 3
10%	4746	4684	4633
20%	13838	13830	13840
30%	23946	23904	23969
40%	34081	33909	34077
50%	42458	42258	42297
60%	47362	47304	47365
70%	49867	49877	49978
80%	51720	51677	51618
90%	52976	53031	53080
100%	52810	52810	52810
