# Supplementary material for: Cell type-specific binding patterns reveal that TCF7L2 can be tethered to the genome by association with GATA3
Source: Genome Biol. 2012 Sep 5;13(9):R52. doi: 10.1186/gb-2012-13-9-r52 (PMC3491396; doi:10.1186/gb-2012-13-9-r52)
Supplement: Additional file 11 — Figure S3 - ChIP-qPCR validation of TCF7L2 sites. [file gb-2012-13-9-r52-S11.pdf]

Figure S3. ChIP-qPCR

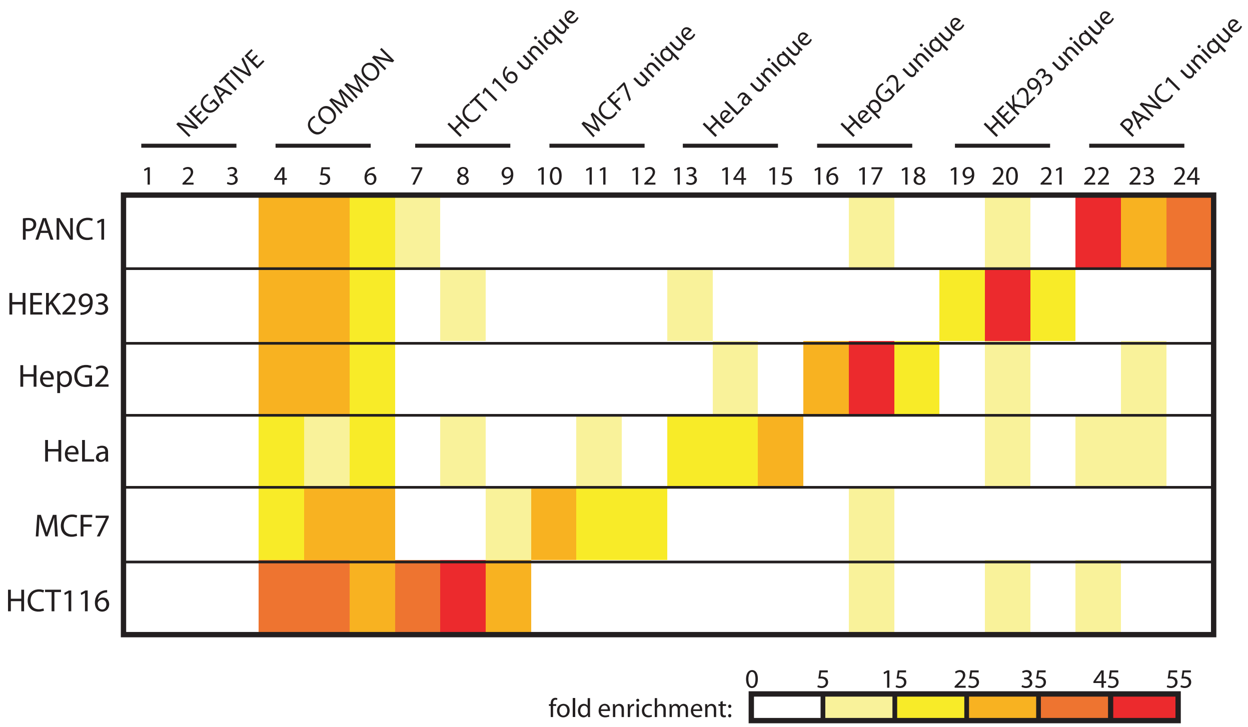

Supplementary Figure S3. ChIP-qPCR validation of TCF7L2 binding sites in different cell lines. TCF7L2 binding sites were identified from ChIP-seq data using the BELT peak-calling program with stringent parameters (see Methods). To identify cell type-specific and common binding sites, peak lists were compared for each cell line. To confirm the TCF7L2 ChIP-seq targets, ChIP-qPCR assays were performed using primers for both common and cell type-unique TCF7L2 targets with ChIP samples that were distinct from the ones analyzed by ChIP-seq. Shown in the heatmap diagram is the average fold enrichment over input for each target as determined by qPCR of two independent ChIP experiments. Primers used in these experiments can be found in Supplementary Table S5.
