# Supplementary material for: Cell type-specific binding patterns reveal that TCF7L2 can be tethered to the genome by association with GATA3
Source: Genome Biol. 2012 Sep 5;13(9):R52. doi: 10.1186/gb-2012-13-9-r52 (PMC3491396; doi:10.1186/gb-2012-13-9-r52)
Supplement: Additional file 12 — Figure S4 - TCF7L2 binds to cell type-specific enhancer regions. [file gb-2012-13-9-r52-S12.pdf]

Figure S4

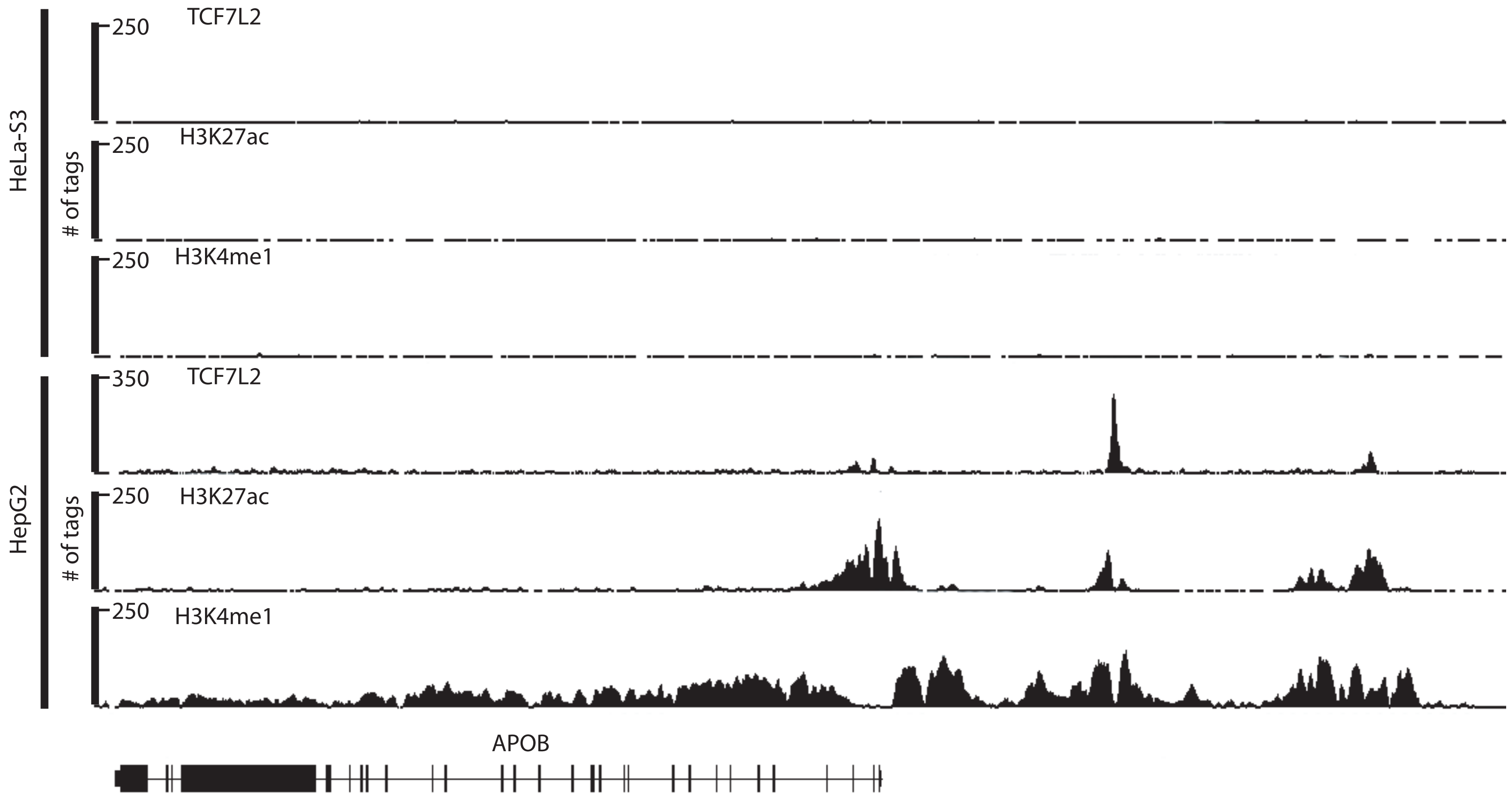

Supplementary Figure S4. TCF7L2 binds to cell type-specific enhancer regions. Binding patterns of TCF7L2, H3K27Ac, and H3K4me1 near the APOB locus are shown for HepG2 cells and HeLa cells; the number of tags reflecting the ChIP enrichments are plotted on the y-axis. The genomic coordinates shown are: chr2: 21,220,000-21,340,000 (hg19).
