# Supplementary material for: Cell type-specific binding patterns reveal that TCF7L2 can be tethered to the genome by association with GATA3
Source: Genome Biol. 2012 Sep 5;13(9):R52. doi: 10.1186/gb-2012-13-9-r52 (PMC3491396; doi:10.1186/gb-2012-13-9-r52)
Supplement: Additional file 13 — Table S6 - TCF7L2 binding motifs in six cell types. We used our ChIPMotifs program to identify two canonical TCF7L2 motifs, W1 of 6 bp and W2 of 8 bp, for each cell type. We then used each of two motifs' position weight matrices to scan the sequences of the peaks to determine how many peaks contained the motifs; we examined the set of all peaks and the set of cell type-specific peaks for all six cell types. [file gb-2012-13-9-r52-S13.pdf]

## Table S6. TCF7L2 binding motifs

| | W1 | percentage | W2 | percentage |
|---|---|---|---|---|
| **HCT116** | | | | |
| all sites (30,266) | **CTTTGA** | 43 | **CCTTTGAT** | 20 |
| cell-specific sites (11,012) | | 37 | | 16 |
| **HepG2** | | | | |
| all sites (27,912) | **CTTTGA** | 54 | **CTTTGATC** | 28 |
| cell-specific sites (14,812) | | 54 | | 26 |
| **HEK293** | | | | |
| all sites (24,457) | **CTTTGA** | 59 | **CTTTGATC** | 28 |
| cell-specific sites (12,847) | | 56 | | 26 |
| **PANC1** | | | | |
| all sites (31,744) | **CTTTGA** | 49 | **CTTTGATC** | 15 |
| cell-specific sites (10,203) | | 50 | | 16 |
| **MCF7** | | | | |
| all sites (27,721) | **CTTTGA** | 50 | **CTTTGATC** | 7 |
| cell-specific sites (14,096) | | 46 | | 5 |
| **HeLa** | | | | |
| all sites (14,755) | **CTTTGA** | 54 | **CTTTGATC** | 30 |
| cell-specific sites (4,073) | | 48 | | 25 |
