# Supplementary material for: Cell type-specific binding patterns reveal that TCF7L2 can be tethered to the genome by association with GATA3
Source: Genome Biol. 2012 Sep 5;13(9):R52. doi: 10.1186/gb-2012-13-9-r52 (PMC3491396; doi:10.1186/gb-2012-13-9-r52)

**Supplementary Figure S5**: Motif recovery percentage plots. We used our ChIPMotifs program to identify two canonical TCF7L2 motifs, W1 of 6 bp and W2 of 8 bp for each cell type. We then used each of two motifs' PWMs to scan the sequences of each set of peaks to determine how many contained either of the two motifs. As seen in the plots, for each cell type the percentage of motifs recovered decreases as more peaks are called.

Supplementary Figure S5

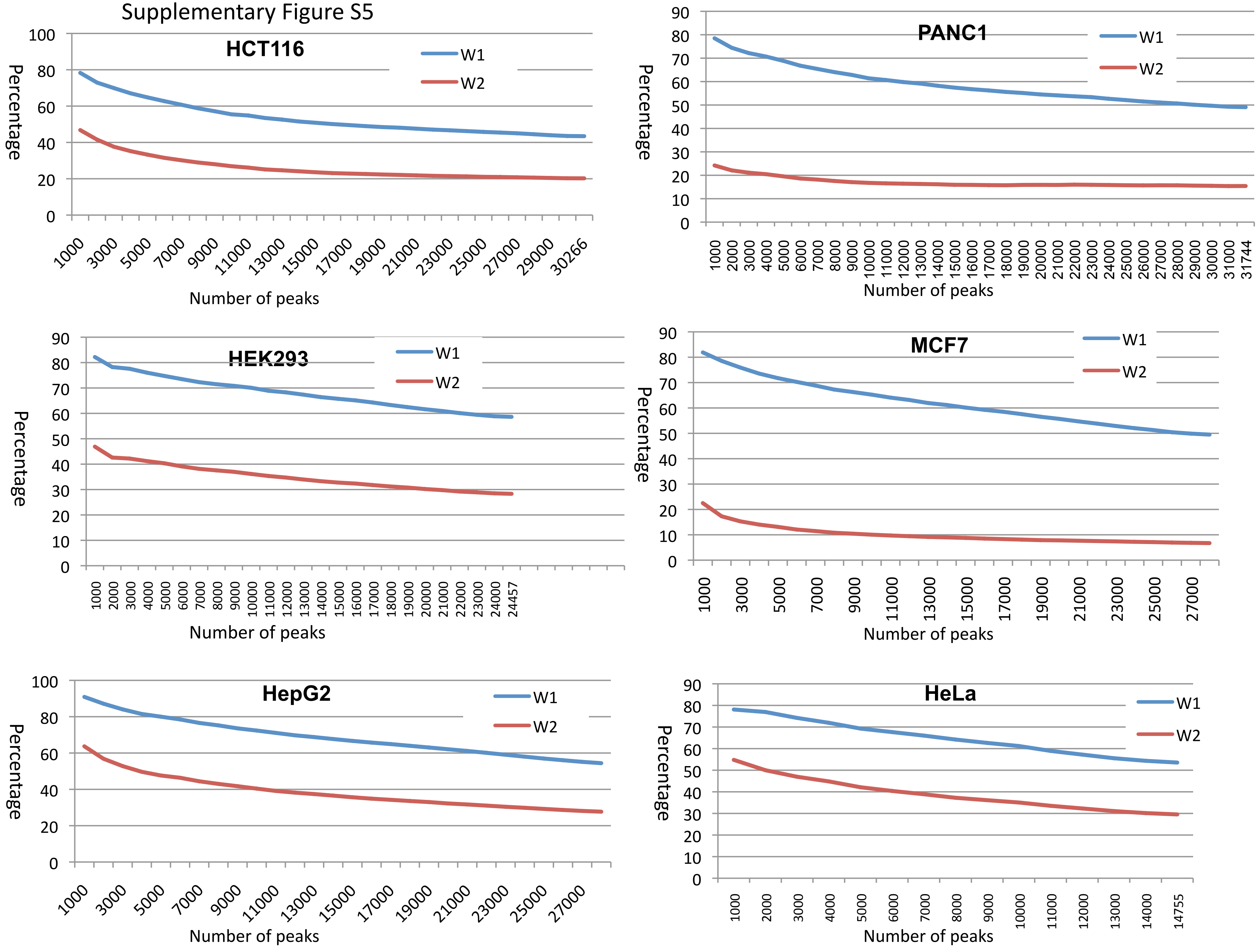

Supplement: Additional file 14 — Figure S5 - motif recovery percentage plots. [file gb-2012-13-9-r52-S14.pdf]
