# Supplementary material for: Cell type-specific binding patterns reveal that TCF7L2 can be tethered to the genome by association with GATA3
Source: Genome Biol. 2012 Sep 5;13(9):R52. doi: 10.1186/gb-2012-13-9-r52 (PMC3491396; doi:10.1186/gb-2012-13-9-r52)
Supplement: Additional file 15 — Figure S6 - Re-ChIP analysis of GATA3 and TCF7L2 sites. [file gb-2012-13-9-r52-S15.pdf]

Supplemental Figure S6

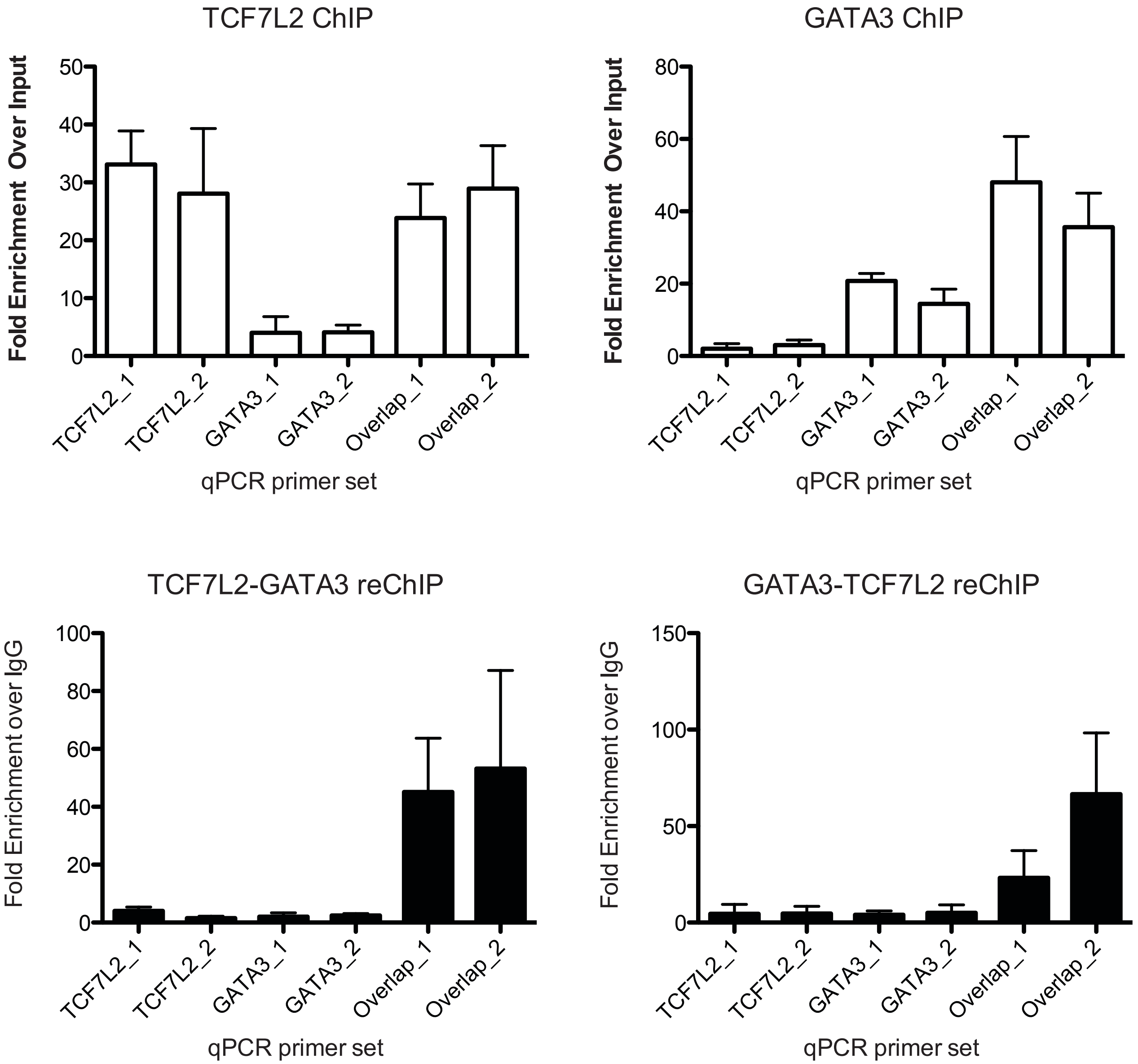

Supplemental Figure 6. ChIP-reChIP assay. TCF7L2 or GATA3 antibodies were used for the first round of ChIP (top panels; white bars), and the order was reversed for the second round of ChIP (bottom panels; black bars). The TCF7L2 and GATA3 individual ChIP assays were normalized against the input. The ChIP-reChIP experiments were normalized against an IgG. We show qPCR performed in triplicate, quantitated and plotted with the standard error of two independent experiments performed. See supplemental methods for experimental details, also see Supplemental Table S5 for qPCR primer sets.
